# The safety of MSC therapy over the past 15 years: a meta-analysis

**DOI:** 10.1186/s13287-021-02609-x

**Published:** 2021-10-18

**Authors:** Yang Wang, Hanxiao Yi, Yancheng Song

**Affiliations:** 1 Department of Orthopedics, The First Affiliated Hospital of Guangdong Pharmaceutical University, Guangdong Pharmaceutical University, No. 19 Nonglinxia Road, Yuexiu District, Guangzhou, Guangdong Province China; 2 Department of Radiotherapy, Sun Yat-Sen Memorial Hospital, Sun Yat-Sen University, No. 600 Tianhe Road, Tianhe District, Guangzhou, Guangdong Province China

**Keywords:** MSC therapy, Randomized clinical trials, All populations, Safety, Meta-analysis

## Abstract

**Background:**

Despite increasing clinical investigations emphasizing the safety of mesenchymal stem cell (MSC) therapy in different populations with different diseases, no article has recently reviewed the adverse events in all populations.

**Aim:**

To evaluate the safety of MSC therapy in all populations receiving MSC therapy and explore the potential heterogeneities influencing the clinical application of MSCs.

**Methods:**

The PubMed, Embase, Web of Science and Scopus databases were searched from onset until 1 March 2021.

**Results:**

All adverse events are displayed as odds ratios (ORs) and 95% CIs (confidential intervals). In total, 62 randomized clinical trials were included that enrolled 3546 participants diagnosed with various diseases (approximately 20 types of diseases) treated with intravenous or local implantation versus placebo or no treatment. All studies were of high quality, and neither serious publication bias nor serious adverse events (such as death and infection) were discovered across the included studies. The pooled analysis demonstrated that MSC administration was closely associated with transient fever (OR, 3.65, 95% CI 2.05–6.49, *p* < 0.01), administration site adverse events (OR, 1.98, 95% CI 1.01–3.87, *p* = 0.05), constipation (OR, 2.45, 95% CI 1.01–5.97, *p* = 0.05), fatigue (OR, 2.99, 95% CI 1.06–8.44, *p* = 0.04) and sleeplessness (OR, 5.90, 95% CI 1.04–33.47, *p* = 0.05). Interestingly, MSC administration trended towards lowering rather than boosting the incidence rate of arrhythmia (OR, 0.62, 95% CI 0.36–1.07, *p* = 0.09).

**Conclusions:**

Conclusively, MSC administration was safe in different populations compared with other placebo modalities.

**Supplementary Information:**

The online version contains supplementary material available at 10.1186/s13287-021-02609-x.

## Introduction

Mesenchymal stromal cells (MSCs), a class of highly heterogeneous cells that can be isolated from bone marrow, adipose tissue, the umbilical cord and the placenta, were primarily discovered in 1974 by Friedenstein [1]. Over the years, exogenous MSCs have amazingly been found to have therapeutic effects in many diseases (e.g. myocardial infarction, liver cirrhosis, limb ischaemia and spinal cord injury) [2–5].

Different from multipotent stem cells, the potency of MSCs is restricted, but MSCs can be induced into osteoblasts, chondrocytes and adipocytes in vitro. Universally, MSCs exert their favourable effects by immunomodulatory regulation and paracrine mechanisms [6, 7]. Clinically, MSCs have been applied in many refractory diseases, such as cerebral palsy [8], spinal cord injury [9] and systemic lupus erythematosus [10]. However, MSCs easily gather together, forming the core of clots and leading to vascular disorders. Additionally, MSCs are tumourigenic as a result of their reproductive capacity and can potentially cause acute or chronic immunogenicity of the cells themselves as foreign matter [11–13]. A large number of studies, most of which have enrolled small samples, have investigated the safety of MSC transplantation, but no articles have reviewed these studies to characterize the adverse events closely associated with MSC administration over the past 9 years.

We performed this meta-analysis to identify all treatment-related adverse events concerning MSC administration and explore the safety of MSCs in clinical utilization.

## Methods and materials

### Search results

This meta-analysis was limited to published articles assessing the safety of MSC administration and was performed by searching the PubMed, EMBASE, Web of Science, Scopus and Cochrane Library databases (from inception to 1 March 2021). The search strategy was as follows: ((MSC [title/abstract]) OR (mesenchymal stem cell [title/abstract]) OR (Wharton’s jelly [title/abstract])) AND ((safety [title/abstract]) OR (side event [title/abstract]) OR (side effect [title/abstract]) OR (adverse event [title/abstract]) OR (adverse effect [title/abstract])). The reference lists of the included articles were also browsed to identify potential studies. To perform a comprehensive search, we did not limit the “study type”; retrospective studies were excluded during the study selection process. The detailed database search strategy is provided in Additional file 1.

### Article selection

Primarily, duplicates of any articles were excluded. Two participants initially screened all titles and abstracts to preclude articles unrelated to our research objectives. Then, we carefully read the full manuscripts and selected the eligible manuscripts.

### Eligibility criteria

The selection process strictly obeyed the PICOS (participants, interventions, comparison, outcome and study) principles, which are listed in Table 1.

**Table 1:** Inclusion and exclusion principles

Principle	Inclusion criteria	Exclusion criteria
Population	Any populations including the healthy people and the diseased people	NA
Intervention	Using MSC as treatment, regardless of the administration methods (e.g. local implantation and injection) and sources of MSC (e.g. from the adipose, bone marrow and gum)	Using NSC, ESC, olfactory neuron, Schwann cell, h-IPS and stem cell from body fluids (e.g. saliva, urine, serum and tears), etc. but MSC as interventions
Comparison	Placebo treatment, nontreatment or basic treatment both utilized in the control and the intervention groups	Merely using traditional treatment (surgery and drug) in the control group but not in the intervention group
Outcome	(1) Any side events associated with MSC treatment; (2) one side event reported by more than one study; (3) regardless of the efficacy of MSC therapy for any diseases	No side events reported
Study	(1) RCT; (2) prospective controlled study	(1) Case report (series); (2) single-arm study; (3) retrospective controlled study; (4) cross-controlled study; (5) study protocol

*ESC* embryonic stem cell, *MSC* mesenchymal stromal cells, *NSC* neuronal stem cell, *h-IPS* human-induced pluripotent stem cell, *NA* not available

### Data extraction

Two skilled reviewers (YW and HXY) independently extracted data from all of the articles according to preset criteria. We retrieved 12 characteristic entries from the original articles, including author, year, study type, location, disease, cell type, administration method, study phase, language, dose, follow-up day and number of patients in each group. Conflicts were resolved in consultation with a third referee.

### Quality assessment

Risk of bias in individual studies and across studies was performed using the Cochrane Collaboration’s tool for assessing the risk of bias.

### Outcome definition

In total, we reported 17 adverse events that appeared during MSC therapy, of which 9 events (death, infection, diarrhoea, central nervous system disorders, arrhythmia, urticaria/dermatitis, vascular disorders, fever, administration site adverse events) were classified as major events, and 8 events (anaemia, constipation, metabolism disorders, fatigue, nausea, seizure, sleeplessness and vomiting) were classified as minor events. One event was considered a major event if it was reported by more than 5 studies or was life-threatening as judged by our clinician; otherwise, it was classified as a minor event. Among these events, some events were not specifically clinical symptoms but referred to a series of correlated symptoms, such as central nervous system disorders, vascular disorders, infections, arrhythmias, administration site adverse events, and metabolism and nutrition disorders. These adverse events are redefined in Table 2. Other entries were retrieved from the original definitions.

**Table 2:** Outcome definition

Side event	Definition
Vascular disorders	(1) Vascular thrombosis including venous and arterial thrombosis; (2) vasculitis
Arhythmia	(1) PSVT; (2) VT; (3) atrial fibrillation; (4) ventricular fibrillation
Central nervous disorders	(1) dizzy; (2) headache
Diarrhoea	Noninfectious diarrhoea
Infection	(1) Noninjection site infection; (2) respiratory system infection; (3) urinary system infection; (4) biliary tract; (5) digestive tract and spontaneous peritonitis
Fever	Transient fever (low-grade, 37.3–38 °℃) within 48 h
Administration site conditions	(1) Injection site bleeding; (2) injection site swelling; (3) injection site pain; (4) injection site itchy; (5) injection site infection
Anaemia	Defined by Hb < 110 g/L
Metabolism and nutrition disorders	Mainly refer to malnutrition

*PSVT* paroxysmal supraventricular tachycardia, *VT* ventricular tachycardia

### Statistical analysis

All of the data were synthesized using R software, version 4.0.3 (University of Auckland, New Zealand). All of the results presented in this article are presented as odds ratios (ORs) with 95% CIs for outcomes. A random-effects model was used to analyse the data when heterogeneity was significant (*p* < 0.05 or *I*^2^ > 50%); otherwise, a fixed-effects model was used. Egger’s and Begg’s tests were utilized to analyse the publication bias of the included articles with R software, version 4.0.3 (meta-package). Subgroup analysis was also conducted to identify potential heterogeneous factors.

## Results

The items of this meta-analysis were reported according to the Preferred Reporting Items for Systematic Review and Meta-Analysis (PRISMA) guideline (Additional file 2).

### Article selection process

**Fig. 1:**
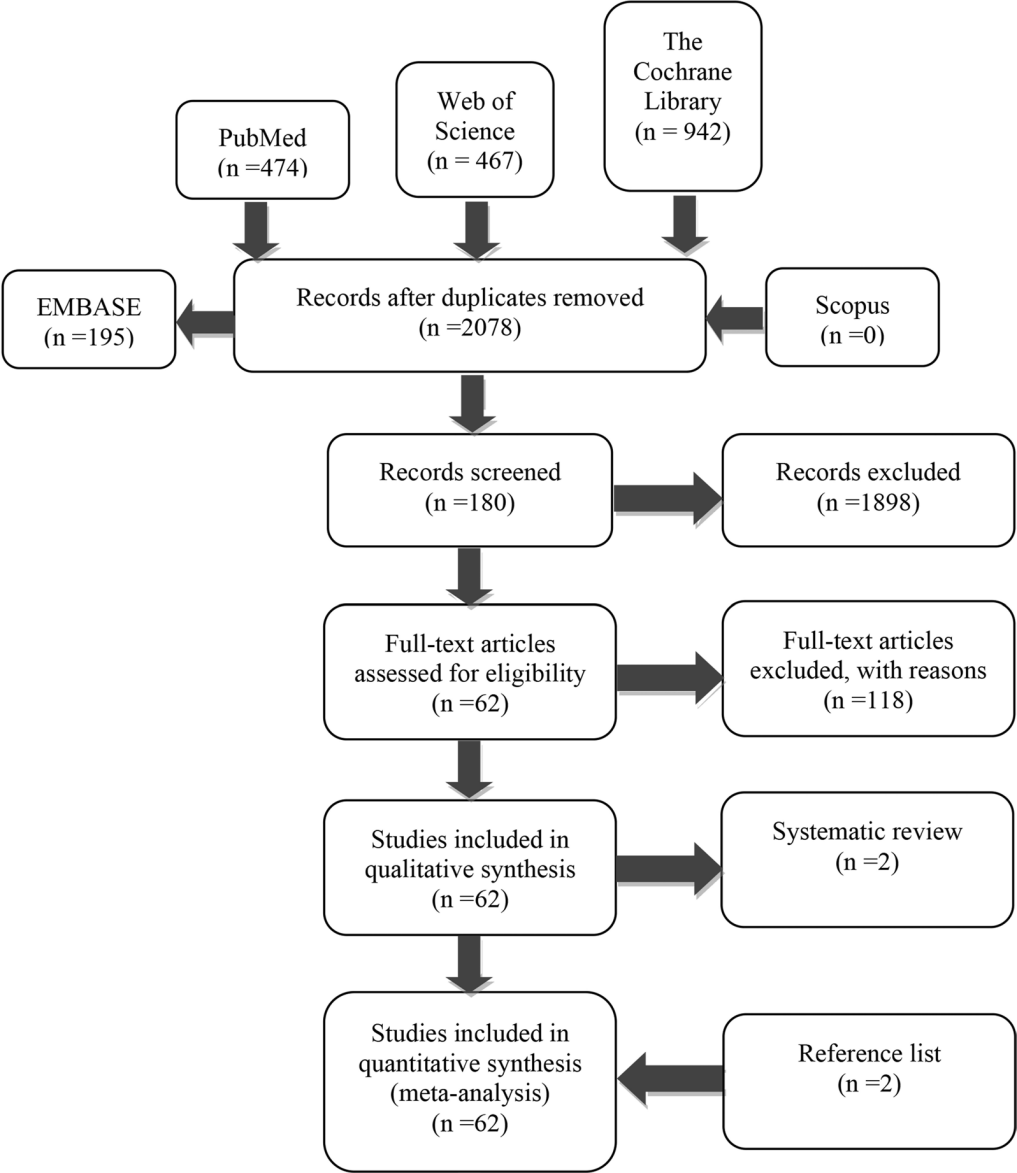
Article selection process

Approximately 2078 articles were identified after the initial search. A total of 1898 irrelevant articles were eliminated by browsing titles and abstracts, and 118 articles were excluded due to unexpected outcomes and interventions. Finally, 62 clinical trials, including 2 trials from reference lists, were included in the analysis despite the elimination of 2 systematic reviews (Fig. 1).

### Baseline of included studies

The data were extracted from studies performed over the past 11 years. Only 2 studies were prospective, nonrandomized trials, and the rest were randomized, controlled trials (RCTs) ranging from study phase 1/2 to study phase 3. Asia was ranked first with the largest number of studies, followed by North America and Europe. The MSCs used in these studies were mainly isolated from bone marrow, adipose tissue and umbilical cords. The injection doses ranged from 4 × 10^7^ to 1.2 × 10^9^ cells. The follow-up length was from 6 months to 2 years (Table 3).

**Table 3:** Study characteristics

Author	Year	Study type	Location	Disease	Cell	Administration	Analysis	Study phrase	Language	Dose	Follow-up
Emadedin	2018	RCT	Iran	Knee osteoarthritis	BMSC	Local implanation	PP	1/2	English	40 × 10^6^ cells	6 months
Gao	2013	RCT	China	Acute myocardial infarction	BMSC	Intracoronary injection	PP	3	English	(3.08 ± 0.52) × 10^6^ cells	1 year
Gupta	2013	RCT	India	Critical limb ischemia	BMSC	Local injection	ITT	1/2	English	2 × 10^6^ cells/kg	2 years
Lee	2010	RCT	South Korea	Ischemic stroke	BMSC	Intravenous injection	PP	3	English	1 × 10^8^ cells	5 years
Zollino	2018	RCT	Italy	Chronic leg ulcers	BMSC	Local injection	PP	2	English	NA	24 weeks
Weiss	2013	RCT	USA	COPD	BMSC	Intravenous injection	PP	3	English	100 × 10^6^ cells	2 years
Xiao	2017	RCT	China	Dilated cardiomyopathy	BMSC	Intracoronary injection	PP	3	English	(4.9 ± 1.7) × 10^8^ cells	1 year
Hare	2009	RCT	USA	Acute myocardial infarction	BMSC	Intravenous injection	PP	3	English	0.5, 1.6 and 5 × 10^6^ cells/kg	6 months
Huang	2018	RCT	China	Cerebral palsy	uc-MSC	Intravenous injection	PP	3	English	5 × 10^7^ cells	2 years
Centeno	2014	RCT	USA	Knee osteoarthritis	BMSC	local implanation	PP	3	English	75% BMSC, 12.5% PRP and 12.5% PBS	2 years
FernaÂndez	2018	RCT	Spain	Multiple sclerosis	AD-MSC	Intravenous injection	ITT	1/2	English	1 × 10^6^ cells/kg, 4 × 10^6^ cells/kg	1 year
Vangsness	2014	RCT	USA	Partial medial meniscectomy	BMSC	Local implanation	PP	3	English	50 × 10^6^ cells	2 years
Lin	2017	RCT	China	Liver failure	BMSC	Intravenous injection	PP	3	English	1.0–10 × 10^5^ cells/kg	2 years
Molendijk	2015	RCT	USA	Crohn’s disease	BMSC	Local implanation	PP	3	English	1–9 × 10^7^ cells	2 years
Tompkins	2017	RCT	USA	Aging frailty	BMSC	Intravenous injection	PP	2	English	100–200 × 10^6^ cells	6 months
Li	2013	RCT	China	Leg ischemia	BMSC	Local implanation	PP	3	English	1 × 10^7^ cells/mL	6 months
Lee	2007	RCT	South Korea	Multiple system atrophy	BMSC	Intravenous injection	PP	3	English	4 × 10^7^ cells	1 year
Jaillard	2020	RCT	France	Ischemic stroke	BMSC	Intravenous injection	ITT	3	English	100–300 × 10^6^ cells	2 year
Mathiasen	2019	RCT	Denmark	Ischaemic heart failure	BMSC	Intramyocardial injection	ITT	3	English	0.2 mL	1 year
Bartunek	2016	RCT	USA	Ischaemic heart failure	BMSC	Intramyocardial injection	ITT	3	English	24 × 10^6^ cells	39 weeks
Bartunek	2013	RCT	Belgium	Heart failure	BMSC	Intramyocardial injection	PP	3	English	600 -1200 × 10^6^ cells	2 years
Powell	2012	RCT	USA	Critical limb ischemia	BMSC	Local implantation	PP	3	English	0.5 ml	1 year
Olivera	2017	RCT	Brazil	Pulmonary emphysema	BMSC	Intravenous injection	ITT	1	English	10^8^ cells	3 months
Sponer	2018	Prospective study	Czech	Femoral bone defects	BMSC	Local implantation	PP	3	English	(15 ± 4.5) × 10^6^ cells	1 year
Wang	2016	RCT	China	Knee osteoarthritis	uc-MSC	Local implantation	PP	3	Chinese	2–3 × 10^7^ cells	6 months
Teraa	2015	RCT	The Netherlands	Limb ischemia	BMSC	Local implantation	ITT	3	English	144–500 × 10^6^ cells	6 months
Bhansali	2016	RCT	USA	Diabetes mellitus	BMSC	Intravenous injection	PP	3	English	1 × 10^6^ cells /kg	1 year
Panes	2016	RCT	Spain	Crohn’s disease	AD-MSC	Intralesional injection	ITT	3	English	120 × 10^6^ cells	24 weeks
Averyanov	2019	RCT	Russia	Idiopathic pulmonary fibrosis	BMSC	Intravenous injection	PP	3	English	2 × 10^8^ cells	1 year
Skyler	2015	RCT	USA	Type 2 diabetes	BMSC	Intravenous injection	ITT	3	English	0.3–2 × 10^6^ cells/kg	1 year
Shi	2010	RCT	China	Chronic liver failure	BMSC	Intravenous injection	PP	3	English	0.5 × 10^6^ cells/kg	72 weeks
Lublin	2014	RCT	USA	Multiple sclerosis	Placenta-Derived MSc	Intravenous injection	ITT	3	English	600 × 10^6^ cells	1 year
Zhang	2011	RCT	China	Liver cirrhosis	uc-MSC	Intravenous injection	PP	3	English	0.5 × 10^6^ cells/kg	1 year
Winkler	2018	RCT	Germany	Hip arthroplasty	Placenta-derived MSc	Local implantation	ITT	3	English	1.5–3.0 × 10^8^ cells	2 years
Kim	2018	RCT	South Korea	Myocardial infarction	BMSC	Intracoronary injection	PP	3	English	(7.2 ± 0.90) × 10^7^ cells	1 year
Tan	2012	RCT	China	Kidney transplants	BMSC	Intravenous injection	PP	3	English	1–2 × 10^6^/kg	1 year
Hauzeur	2017	RCT	Belgium	Osteonecrosis	BMSC	Local implantation	PP	3	English	50 ml	2 years
Noriega	2017	RCT	Spain	Intervertebral disc regeneration	BMSC	Local implantation	PP	3	English	25 × 10^6^ cells	1 year
Gao	2015	RCT	China	Myocardial infarction	uc-MSC	Intracoronary injection	PP	3	English	6 × 10^6^ cells	18 months
Ascheim	2014	RCT	USA	Left ventricular assist device patients	BMSC	Intramyocardial injection	PP	3	English	25 × 10^6^ cells	1 year
Koh	2012	RCT	South Korea	Knee osteoarthritis	BMSC	Local implantation	PP	3	English	1.89 × 10^6^ cells	1 year
Berry	2019	RCT	USA	Amyotrophic lateral sclerosis	BMSC	Local implantation	PP	2	English	125 × 10^6^ cells	6 months
Chullikana	2014	RCT	India	Acute myocardial infarction	BMSC	Intracoronary injection	PP	1/2	English	6.0 × 10^7^ cells	2 years
Oh	2018	RCT	Republic of Korea	Amyotrophic lateral sclerosis	BMSC	Intrathecal injections	ITT	3	English	1 × 10^6^ cells/kg	6 months
Hess	2017	RCT	USA	Acute ischaemic stroke	BMSC	Intracerebral injection	ITT	2	English	1200 × 10^6^ cells	3 months
Bartolucci	2017	RCT	Chile	Heart failure	uc-MSC	Intravenous injection	PP	1/2	English	1 × 10^6^ cells/kg	1 year
Wang	2017	RCT	Australia	Anterior cruciate ligament reconstruction patients	BMSC	local implantation	ITT	3	English	150 × 10^6^ cells	104 weeks
Wang	2014	RCT	China	Lyocardial infarction	BMSC	Intracoronary injection	PP	3	English	1 × 10^8^ cells/ml	1 month
Gu	2020	RCT	China	Cerebral palsy	uc-MSC	Intravenous injection	PP	3	English	4.5–5.5 × 10^7^ cells	1 year
Zhang	2016	RCT	China	Liver transplantation	uc-MSC	Intravenous infusion	PP	3	English	1.0 × 10^6^ cells/ kg	2 years
Suk	2016	RCT	South Korea	Alcoholic cirrhosis	BMSC	Hepatic arterial injection	PP	2	English	5 × 10^7^ cells	1 year
Zheng	2014	RCT	China	Acute respiratory distress syndrome	AD-MSC	Intravenous infusion	PP	3	English	1 × 10^6^ cells/kg	4 weeks
Matas	2019	RCT	Chile	Knee Osteoarthritis	AD-MSC	Local implantation	PP	1/2	English	2 × 10^7^cells	1 year
Wang	2006	RCT	China	Dilatedcardiomyopathy	BMSC	Intracoronary injection	ITT	3	Chinese	8 × 10^7^ cells	6 months
Lin	2012	RCT	China	Liver fibrosis	uc-MSC	Intravenous infusion	ITT	3	Chinese	0.5–1 × 10^6^ cells/kg	48 weeks
Xie	2007	RCT	China	Spinal cord injury	BMSC	Intrathecal injection	ITT	3	Chinese	20.56–58.87 × 10^8^ cells	3 months
Xiao	2012	RCT	China	Myocardial infarction	BMSC	Intracoronary injection	ITT	3	Chinese	1–10 × 10^9^ cells	3 months
Erpicum	2018	RCT	Belgium	Kidney transplantation	BMSC	Intravenous injection	PP	1/2	English	2 × 10^6^ cells/kg	1 year
Shadmafar	2017	Prospective study	China	Liver fibrosis	uc-MSC	Hepatic artery injection	ITT	3	English	(42 ± 4) × 10^6^ cells	1 year
Zeng	2015	RCT	Iran	Rheumatoid arthritis	BMSC	Local implantation	PP	1/2	Chinese	6.0–7.0 × 10^7^ cells/kg	52 weeks
Ning	2008	RCT	China	Hematologic malignancy	BMSC	Intravenous injection	NA	NA	English	1.0–2.0 × 10^6^ cells/kg	3 years
José	2020	RCT	Spain	Knee osteoarthritis	BMSC	Local implantation	ITT	2	English	100 × 10^6^ cells	1 year

*uc-MSC* human umbilical cord mesenchymal stromal cell, *BMSC* bone marrow-derived human umbilical cord mesenchymal stromal cell, *AD-MSC* adipose-derived human umbilical cord mesenchymal stromal cell, *PP* per-protocol, *ITT* intention to treat, *RCT* randomized clinical trial, *COPD* chronic obstructive pulmonary disease

### Pooled analysis of all studies

**Fig. 2:**
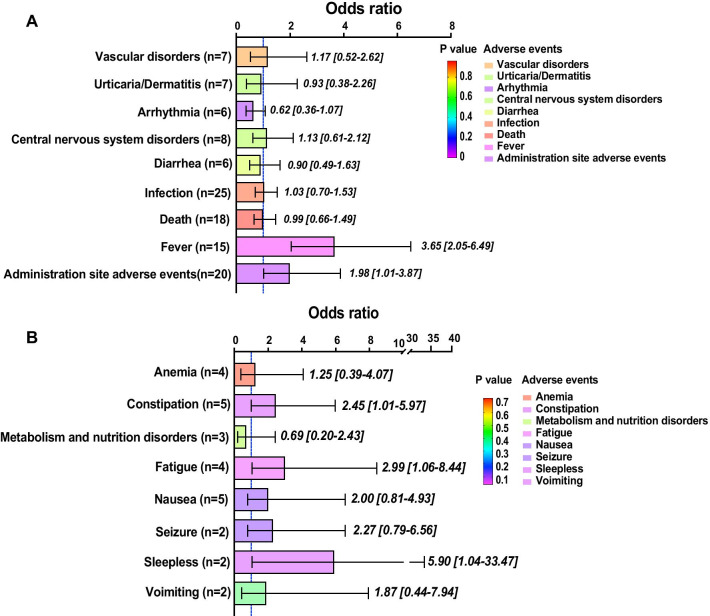
Bar plot for events in all articles. This figure depicts the significance of major events (**A**) and minor events (**B**) in all of the included articles. The OR value of each pooled event is presented as the mean and 95% confidential intervals. The significance of each event is marked by different colours. The closer that the colour approaches the bottom of the *p* value bar, the more significant that the occurrence of the event is. Scarcely reported events (reported by a single article) were not collected and are considered minor events

In total, 62 clinical trials containing populations with different characteristics were included in the analysis (Fig. 2A). We discovered that MSC administration was not closely related to major adverse events, such as vascular disorders (1.17, 95% CI 0.52–2.62, *p* = 0.70), urticaria/dermatitis (0.93, 95% CI 0.93–1.07, *p* = 0.70), central nervous system disorders (1.13, 95% CI 0.61–2.12, *p* = 0.69), diarrhoea (0.90, 95% CI 0.49–1.63, *p* = 0.73), death (0.99, 95% CI 0.66–1.49, *p* = 0.96) or infection (1.03, 95% CI 0.70–1.53, *p* = 0.87). However, our analysis demonstrated that transient fever (3.65, 95% CI 2.05–6.49) possibly occurred within 48 h if people received MSC administration. At the same time, MSC injections also potentially caused adverse events at the administration site (1.98. 95% CI 1.01–3.87, *p* < 0.01). Populations tended to benefit from receiving MSC therapy since they tended to have a lower rate of arrhythmia (0.62, 95% CI 0.36–1.07, *p* = 0.05).

Regarding minor adverse events, MSCs were potentially associated with sleeplessness (5.90, 95% CI 1.04–33.47, *p* = 0.05), constipation (2.45, 94% CI 1.01–5.97, *p* = 0.05) and fatigue (2.99, 1.06–8.44, *p* = 0.05). Other minor adverse events, including anaemia (1.25, 95% CI 0.39–4.07, *p* = 0.71), metabolism and nutrition disorders (0.69, 95% CI 0.20–2.43, *p* = 0.56), nausea (2.00, 95% CI 0.81–4.93, *p* = 0.13), seizures (2.27, 94% CI 0.79–6.56, *p* = 0.13) and vomiting (1.87, 95% CI 0.22–7.94, *p* = 0.40), were nonsignificantly correlated with MSC treatment (Fig. 2B).

### Subgroup analysis of all studies

**Fig. 3:**
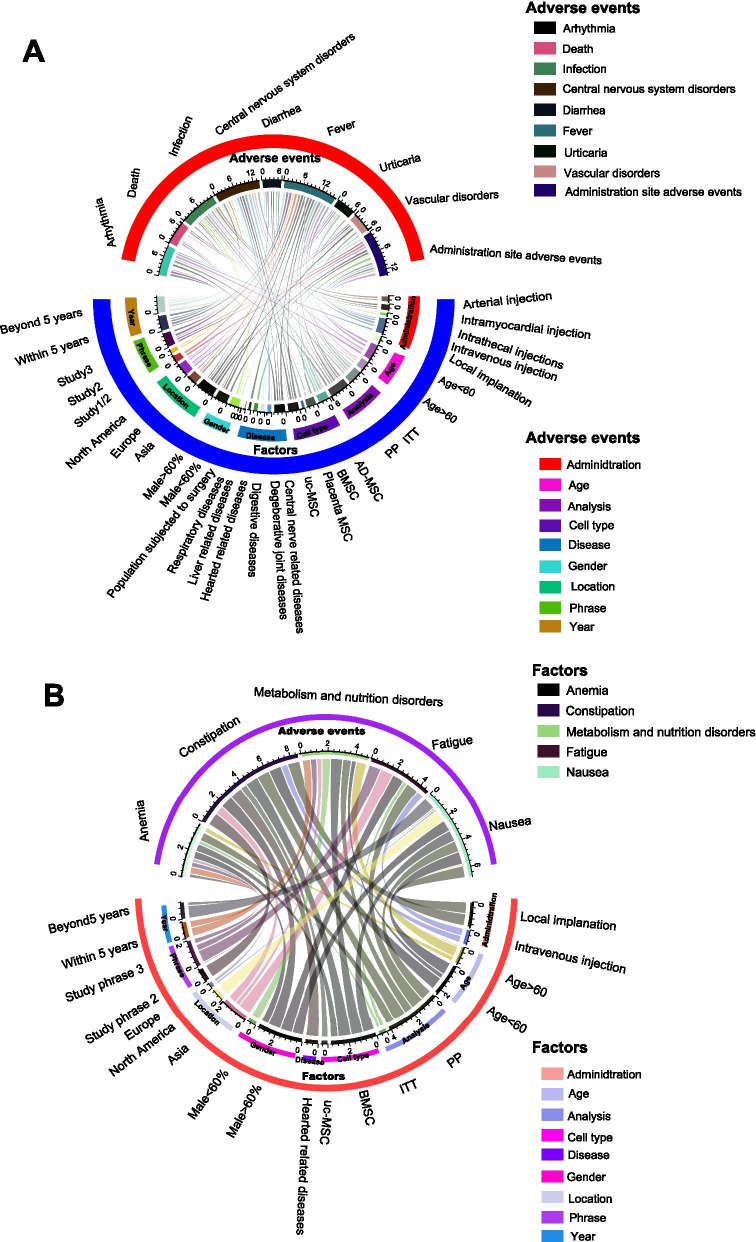
Bar plot for events in high-quality articles. This figure depicts the significance of major events (**A**) and minor events (**B**) in high-quality articles. The OR value of each pooled event is presented as the mean and 95% confidential intervals. The significance of each event is marked by different colours. The closer that the colour approaches the bottom of the *p* value bar, the more significant that the occurrence of the event is. Scarcely reported events (reported by a single article) were not collected and are considered minor events

Subsequently, we extracted potential factors, including administration (method), age, methodology (analysis) of the article, cell type, population (disease), gender proportion, location, study phase and publication date (year), influencing the major adverse events (Fig. 3A). We identified that the nonsignificance of death, infection and diarrhoea, which were not treatment-related adverse events of MSC therapy, were not altered in the slightest by any of the analysed factors. MSC therapy was demonstrated to be correlated with a lower incidence of arrhythmia in the population aged < 60 years old (*p* < 0.01), those undergoing PP analysis (*p* = 0.01) and those treated beyond 5 years (*p* < 0.01). Despite the nonsignificant central nervous system disorders (head and dizziness) proved by pooled analysis, AD-MSCs (*p* < 0.01), placental MSCs (*p* < 0.01) and uc-MSCs (*p* < 0.01) more easily caused headache and dizziness. At the same time, a population with degenerative joint diseases (*p* < 0.01) and digestive diseases (*p* < 0.01) could potentially have headache and dizziness symptoms while receiving MSC implantation. Urticaria significantly occurred when the data were analysed exclusively by PP analysis (*p* < 0.01). Regarding vascular disorders, Asian people tended to have vascular disorders (*p* < 0.01) after MSC treatment. Administration site adverse events preferably occurred in studies with populations with age < 60 years old (*p* = 0.02), populations with heart-related diseases (*p* = 0.01) and male proportions > 60% (*p* = 0.08), in study phase 1/2 (*p* = 0.01), and within 5 years (*p* = 0.05). Although transient fever was conspicuously associated with MSC treatment, populations aged > 60 years old (*p* = 0.86), male proportion < 60% (*p* = 0.7), receiving local implantation (*p* = 0.76), being North American (*p* = 0.82) and phase 1 studies (*p* = 0.15) had a lower risk of transient fever over the period of MSC therapy.

In terms of minor adverse events, only five adverse events, namely anaemia, constipation, metabolism and nutrition disorders and nausea, were analysed (Fig. 3B). Similarly, the interactions between the 9 predicted factors and seldom reported adverse events were analysed. Contrary to the pooled analysis, neither constipation nor fatigue was a significant adverse event in these subgroup analyses. Similar to the pooled analysis, both metabolism and nutrition disorders and nausea were not impacted by these factors and were nonsignificant adverse events. Interestingly, we found that populations aged < 60 years old tended to have transient anaemia (*p* = 0.07) post-MSC treatment.

### Pooled analysis of high-quality studies

**Fig. 4:**
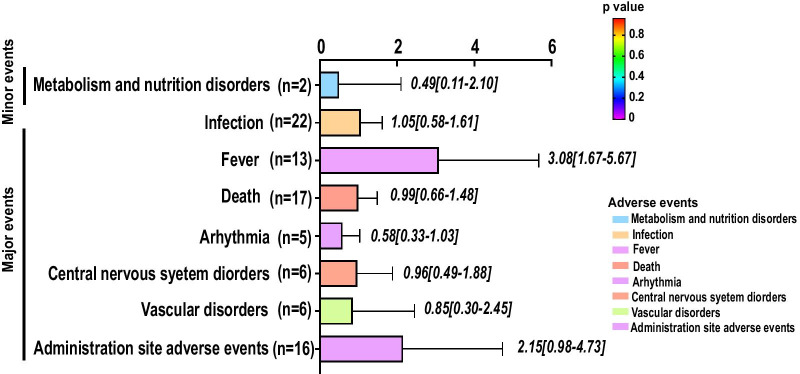
Circular network map for events of all articles. This figure depicts potential factors impacting major events (**A**) and minor events (**B**) in the included articles. Each line connecting 2 colour blocks indicates a potential interaction between the adverse event and the factor. If no interaction existed between the factor and the adverse event, the value was denoted as 1 by default. The area of the connecting line is proportional to the value of 1 minus *p*. The larger that the connecting line area is, the more likely that the adverse event is to be impacted by the factor

After the elimination of low-quality articles (Kim 2018; Koh 2012; Lee 2017; Lin 2012; Oh 2018; Shi 2012; Sponer 2018; Wang 2006; Wang 2014; Wang 2016; Xie 2007; Zeng 2015; Xiao 2012; Skyler 2015), only seven major adverse events and one minor adverse event remained (Fig. 4). We found a close relationship between transient fever (3.08, 95% CI 1.67–1.48, *p* = 0.01) and MSC administration. Other adverse events, such as metabolism and nutrition disorders (0.49, 95% CI 0.11–2.10, *p* = 0.33), infection (1.05, 95% CI 0.59–1.61, *p* = 0.83), death (0.99, 95% CI 0.66–1.48, *p* = 0.96), arrhythmia (0.58, 95% CI 0.33–1.03, *p* = 0.06), central nervous system disorders (0.96, 95% CI 0.49–1.88, *p* = 0.91), vascular disorders (0.85, 95% CI 0.30–2.45, *p* = 0.77) and administration site adverse events (2.15, 95% CI 0.98–4.73, *p* = 0.06), were not significantly impacted by MSC administration.

### Subgroup analysis of high-quality studies

**Fig. 5:**
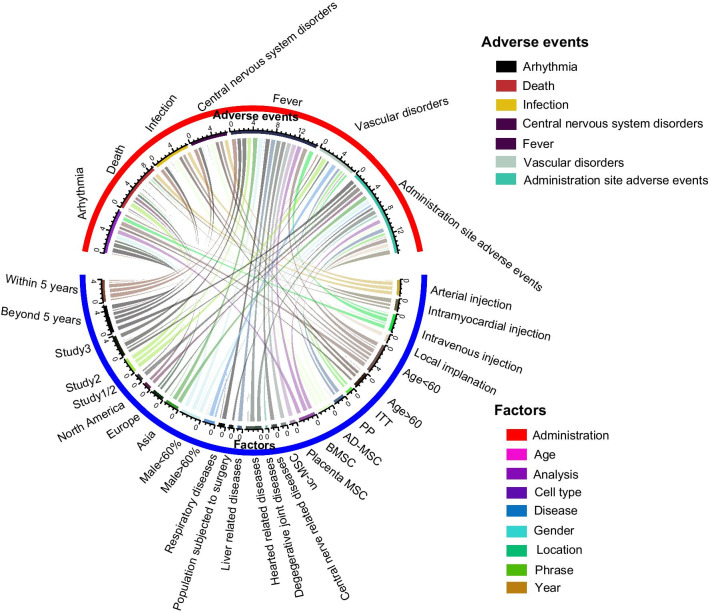
Circular network map for events of high-quality articles. This figure depicts potential factors impacting major events (**A**) and minor events (**B**) in high-quality articles. Each line connecting 2 colour blocks indicates a potential interaction between the adverse event and the factor. If no interaction existed between the factor and the adverse event, the value was denoted as 1 by default. The area of the connecting line is proportional to the value of 1 minus *p*. The larger that the connecting line area is, the more likely that the adverse event is to be impacted by the factor

We examined whether potential factors significantly influenced the terminal outcomes (7 major adverse events) reported by high-quality studies (Fig. 5). MSC administration does not directly lead to death, death, central nervous disorders (headache and dizziness) or vascular disorders. Populations aged < 60 years old (*p* < 0.01), those receiving BMSC injection (*p* = 0.04), those in study phase 3 (*p* = 0.04) and those treated beyond 5 years (*p* < 0.01) seemed to have a lower incidence of arrhythmia and benefit from MSC administration. Regarding transient fever, MSC administration did not trigger fever in populations aged > 60 years old (*p* = 0.86), those with male proportions < 60% (*p* = 0.70), Europeans (*p* = 0.82), phase 2 patients (*p* = 0.15), patients older than 5 years (*p* = 0.11) or patients receiving local implantation (*p* = 0.76).

### Sensitivity analysis

Leave-one-out meta-analysis was performed for administration site adverse events, arrhythmia, death, dermatitis, diarrhoea, transient fever, infection, central nervous system disorders, vascular disorders, fatigue, metabolism and nutrition disorders, anaemia, constipation and nausea from all studies (Additional files 3–16) and for administration site adverse events, arrhythmia, death, transient fever, infection, central nervous system disorders and vascular disorders from high-quality studies (Additional file 17–23).

### Publication bias and article quality

**Fig. 6:**
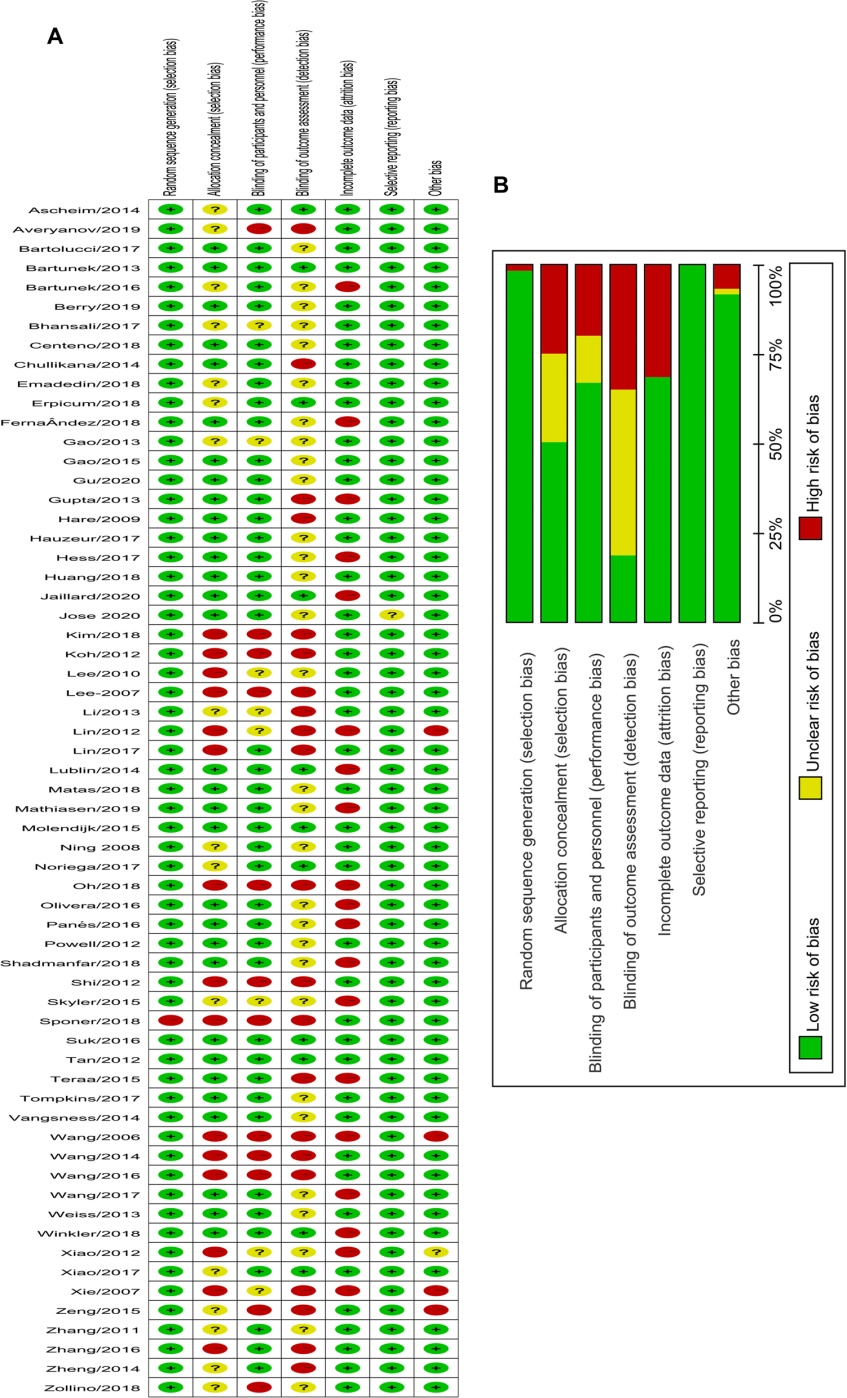
Quality assessment of included articles. **A** Quality assessment of each article. **B** Pooled result of quality assessment

We assessed the article quality using the Cochrane Collaboration’s tool for assessing the risk of bias (Fig. 6). We concluded that most study designs were suitable and of high quality. Only 14 studies were considered low quality because they had more than two entries marked as high risk and fewer than four entries evaluated as low risk. There were performance bias, selection bias, detection bias and attrition bias potentially lowering the integral quality of the included studies. Furthermore, we tested the publication bias for administration site adverse events, arrhythmia, death, dermatitis, diarrhoea, transient fever, infection, central nervous system disorders and vascular disorders (Additional files 24–32) from all of the studies. Publication bias for administration site adverse events, arrythmia, death, fever, infection, central nervous system disorders and vascular disorders (Additional files 33–39) from high-quality studies was also conducted.

## Discussion

### Summary of evidence

The association between adverse events and MSC administration was first reported by Lalu [14], and the associations of MSC administration with infusional toxicity, organ system complications, infection and death were not explored due to limited clinical research. However, aside from the adverse events above, which were analysed in this meta-analysis, more adverse events have been described in recent trials with the expansion of the population. In addition to transient fever, which is the most frequently reported event by researchers, other adverse events, such as constipation, fatigue, administration site adverse events and sleeplessness, can also be induced by MSC administration. For arrhythmia, MSCs seemed to benefit patients with cardiac diseases.

We were unable to detect conspicuous associations between MSC administration and the remaining adverse events (vascular disorders, urticaria/dermatitis, dizziness/headache, diarrhoea, infection, death, anaemia, metabolism and nutrition disorders, nausea, seizure and vomiting), nor was there direct proof suggesting that MSC administration was tumourigenic. To date, malignancy of MSCs has been reported only by Ning [15] despite their potential tumourigenesis.

After the elimination of the low-quality studies, eight adverse events were analysed: including metabolism and nutrition disorders, infection, fever, death, arrhythmia, dizziness/headache, vascular disorders and administration site effects. Among these adverse events, transient fever was exclusively associated with MSC administration. Arrhythmia and administration site adverse events tended to be significant after MSC administration. Other adverse events had no relevance to MSC administration.

Furthermore, we analysed each adverse event in various subpopulations to identify how adverse events were determined. We discovered that age, sex proportion, location, year, analysis, disease, study phase, cell type and administration method were the main factors impacting the final adverse events. Considering the definite adverse event of fever as an example, the elderly were not impacted by MSC administration, perhaps because of blunt reactions of the organism to acute inflammation triggered by MSCs [16]. Women more easily suffer from transient fever, and the oestrogen level is under serious doubt [17]. The population in North America underwent transient fever less often than other regional populations, which could suggest racial discrepancies in MSC administration.

### Strengths and weaknesses

This meta-analysis removed studies with low-grade evidence (retrospective studies, single-arm studies and case studies) and included 62 prospective studies. All of the results suggested strong associations of MSC administration with transient fever and administration site adverse events. Moreover, more adverse events that were not reported before (e.g. anaemia constipation and vomiting) are gradually being discovered [18–20]. Theoretically, the adverse events of MSC administration should be under stringent surveillance in cases of the occurrence of other adverse events that were not reported earlier, along with the expansion of clinical trials. We also noted that the longest follow-up was 5 years, which might be a shorter time considering that we are using cell products. We should be cautious that longer-term events in the future might be impeded.

Our research has limitations. First, we synthesized the data across heterogeneous disease states. Despite subgroup analysis of disease, it was difficult to distinguish whether one adverse event was specifically disease-related owing to the limited number of studies. Second, some studies presented their data in the form of abstracts prior to formal publication, which might have imposed an unknown effect on the interpretation of the outcomes. These data are difficult for us to obtain because many ongoing trials are in the middle stages and the investigators not want to release these data. Third, several adverse events were merely comprehensive conceptions rather than specific clinical symptoms, and we contend that it was important to record these obscure descriptions (e.g. metabolism and nutrition disorders and gastrointestinal dysfunction). Fourth, we were not informed whether the cell dose was closely associated with these adverse events as a result of the lack of dose-dependent trials. If possible, a Bayesian network meta-analysis should be conducted to investigate this point further. Finally, tumourigenesis, which theoretically exists in MSC therapy, has rarely been reported by researchers. This interesting point should draw our attention.

The most clear adverse event discovered by this research was fever, which was presumably caused by the immunoregulatory actions of MSCs, and patients should be informed of this adverse event. Moreover, the security of mass production and uniform quality of MSCs should be resolved if we want the large-scale application of MSCs. Moreover, if we can find a better substitute (a noncell agent) for MSCs, such as MSC-derived microvesicles, which potentially share equal treatment effects as MSCs, the adverse events reported by our research might be of less concern.

## Conclusions

We summarized all adverse events potentially related to the application of MSCs, and no serious safety events other than transient fever, administration site adverse events, sleeplessness and constipation were discovered. Many population characteristics, including age, analysis, cell type, disease, sex, location, study phase, year and administration method, possibly impacted the occurrence of one adverse event. The safety of MSC administration should be assured under sustained observation despite the innovative therapy appearing safe.

## Supplementary Information

**Additional file 1**. Detailed search strategy.

**Additional file 2**. PRISMA 2009 checklist.

**Additional file 3**. Leave-one-out meta-analysis for administration site adverse events.

**Additional file 4**. Leave-one-out meta-analysis for arrhythmia.

**Additional file 5**. Leave-one-out meta-analysis for death.

**Additional file 6**. Leave-one-out meta-analysis for dermatitis.

**Additional file 7**. Leave-one-out meta-analysis for diarrhoea.

**Additional file 8**.Leave-one-out meta-analysis for transient fever.

**Additional file 9**. Leave-one-out meta-analysis for infection.

**Additional file 10**. Leave-one-out meta-analysis for central nervous system disorders.

**Additional file 11**. Leave-one-out meta-analysis for vascular disorders.

**Additional file 12**. Leave-one-out meta-analysis for fatigue.

**Additional file 13**. Leave-one-out meta-analysis for metabolism and nutrition disorders.

**Additional file 14**. Leave-one-out meta-analysis for anaemia.

**Additional file 15**. Leave-one-out meta-analysis for constipation.

**Additional file 16**. Leave-one-out meta-analysis for nausea.

**Additional file 17**. Leave-one-out meta-analysis of administration site adverse events in high-quality studies.

**Additional file 18**. Leave-one-out meta-analysis for arrhythmia in high-quality studies.

**Additional file 19**. Leave-one-out meta-analysis for death in high-quality studies.

**Additional file 20**. Leave-one-out meta-analysis for transient fever in high-quality studies.

**Additional file 21**. Leave-one-out meta-analysis for infection in high-quality studies.

**Additional file 22**. Leave-one-out meta-analysis of central nervous system disorders in high-quality studies.

**Additional file 23**. Leave-one-out meta-analysis for vascular disorders in high-quality studies.

**Additional file 24**. Funnel plot for administration site adverse events.

**Additional file 25**. Funnel plot for arrhythmia.

**Additional file 26**. Funnel plot for death.

**Additional file 27**. Funnel plot for dermatitis.

**Additional file 28**. Funnel plot for diarrhoea.

**Additional file 29**. Funnel plot for transient fever.

**Additional file 30**. Funnel plot for infection.

**Additional file 31**. Funnel plot for central nervous system disorders.

**Additional file 32**. Funnel plot for vascular disorders.

**Additional file 33**. Funnel plot for administration site adverse events in high-quality studies.

**Additional file 34**. Funnel plot for arrythmia in high-quality studies.

**Additional file 35**. Funnel plot for death in high-quality studies.

**Additional file 36**. Funnel plot for fever in high-quality studies.

**Additional file 37**. Funnel plot for infection in high-quality studies.

**Additional file 38**. Funnel plot for central nervous system disorders in high-quality studies.

**Additional file 39**. Funnel plot for vascular disorders in high-quality studies.

## Data Availability

Not applicable.

## Abbreviations

OR: Odds ratio
CI: Confidential intervals
RCTs: Randomized clinical trials
MSCs: Mesenchymal stromal cells
PICOS: Participants, interventions, comparison, outcome and study
NO.: Number
PRISMA: Systematic review and meta-analysis
pp: Per-protocol
ESC: Embryonic stem cell
NSC: Neuronal stem cell
h-IPS: Human-induced pluripotent stem cell
PSVT: Paroxysmal supraventricular tachycardia
VT: Ventricular tachycardia
COPD: Chronic obstructive pulmonary disease
BMSC: Bone marrow-derived human umbilical cord mesenchymal stromal cell
AD-MSC: Adipose-derived human umbilical cord mesenchymal stromal cell
NA: Not available
