# Supplementary material for: The safety of MSC therapy over the past 15 years: a meta-analysis
Source: Stem Cell Res Ther. 2021 Oct 18;12:545. doi: 10.1186/s13287-021-02609-x (PMC8522073; doi:10.1186/s13287-021-02609-x)
Supplement: Supplementary file 1 — Additional file 1. Detailed search strategy. [file 13287_2021_2609_MOESM1_ESM.docx]

**Additional file 1. The detailed search strategy**

| **Databases** | **Search strategy** | **Results** |
| --- | --- | --- |
| **PubMed** | ((MSC [title/abstract]) OR (mesenchymal stem cell [title/abstract]) OR (Wharton’s jelly [title/abstract])) AND ((safety [title/abstract]) OR (side event [title/abstract]) OR (side effect [title/abstract]) OR (adverse event [title/abstract]) OR (adverse effect [title/abstract])) | **474** |
| **Web of Science** |  | **467** |
| **The Cochrane Library** |  | **942** |
| **EMBASE** |  | **195** |
| **Scopus** |  | **0** |
