# Supplementary figures and images for: The safety of MSC therapy over the past 15 years: a meta-analysis
Source: Stem Cell Res Ther. 2021 Oct 18;12:545. doi: 10.1186/s13287-021-02609-x (PMC8522073; doi:10.1186/s13287-021-02609-x)

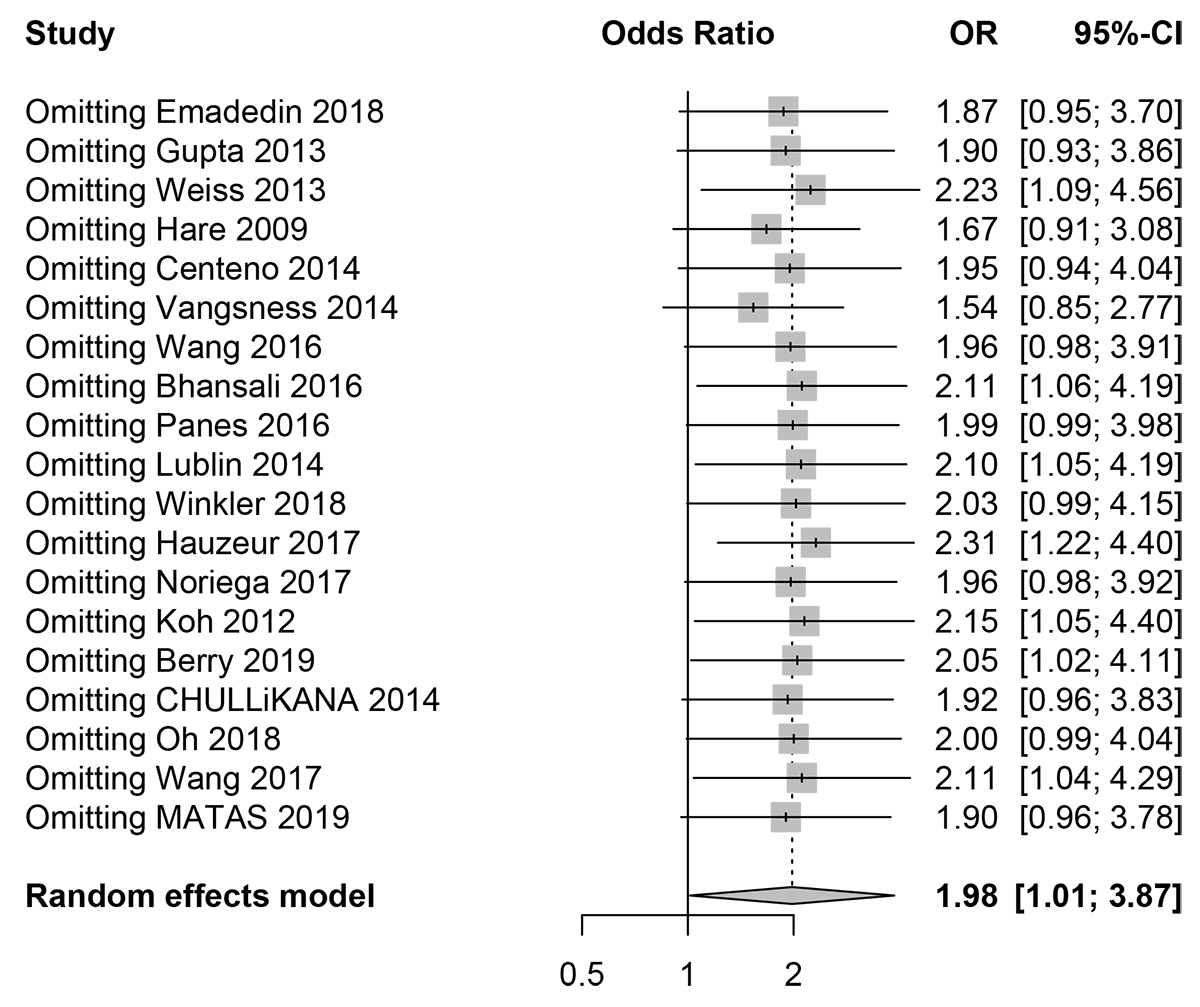

Supplement: Supplementary file 3 — Additional file 3. Leave-one-out meta-analysis for administration site adverse events. [file 13287_2021_2609_MOESM3_ESM.tif]

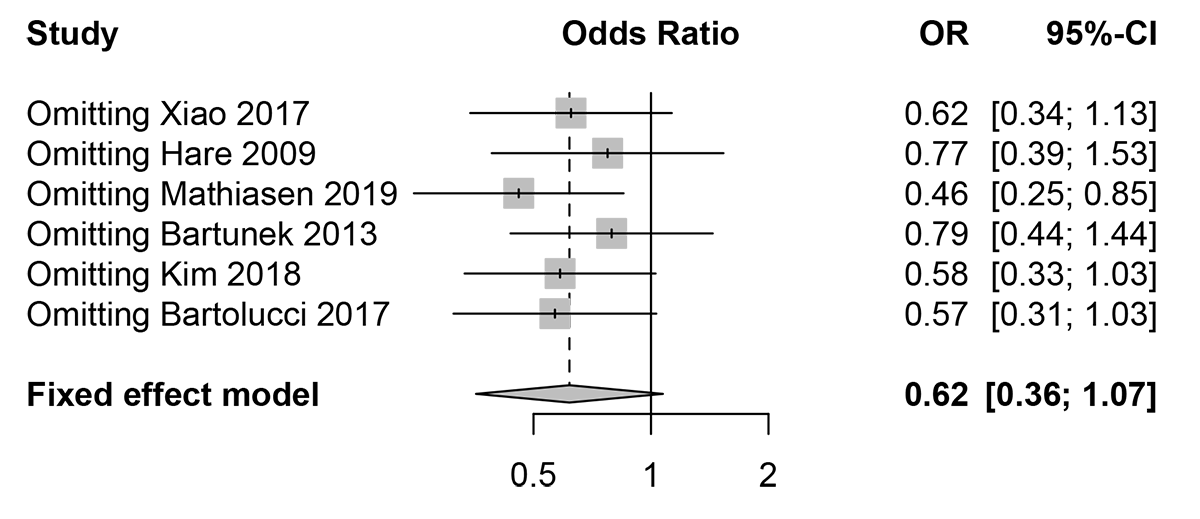

Supplement: Supplementary file 4 — Additional file 4. Leave-one-out meta-analysis for arrhythmia. [file 13287_2021_2609_MOESM4_ESM.tif]

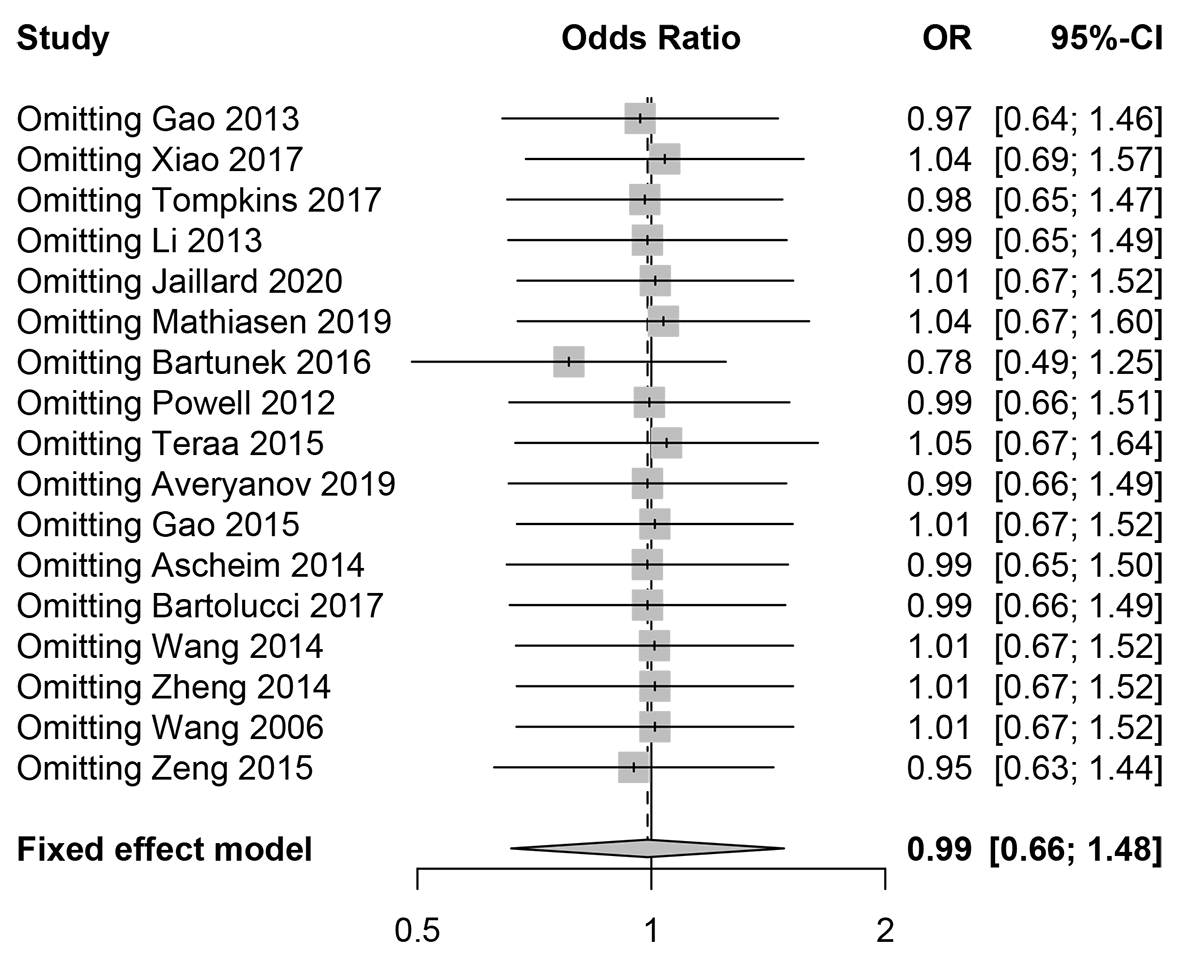

Supplement: Supplementary file 5 — Additional file 5. Leave-one-out meta-analysis for death. [file 13287_2021_2609_MOESM5_ESM.tif]

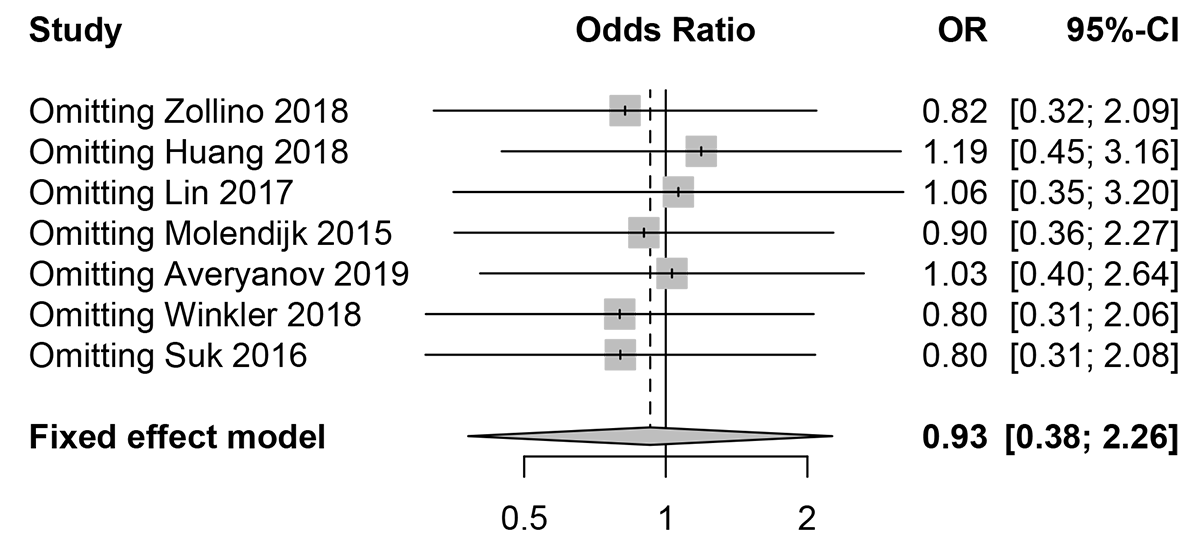

Supplement: Supplementary file 6 — Additional file 6. Leave-one-out meta-analysis for dermatitis. [file 13287_2021_2609_MOESM6_ESM.tif]

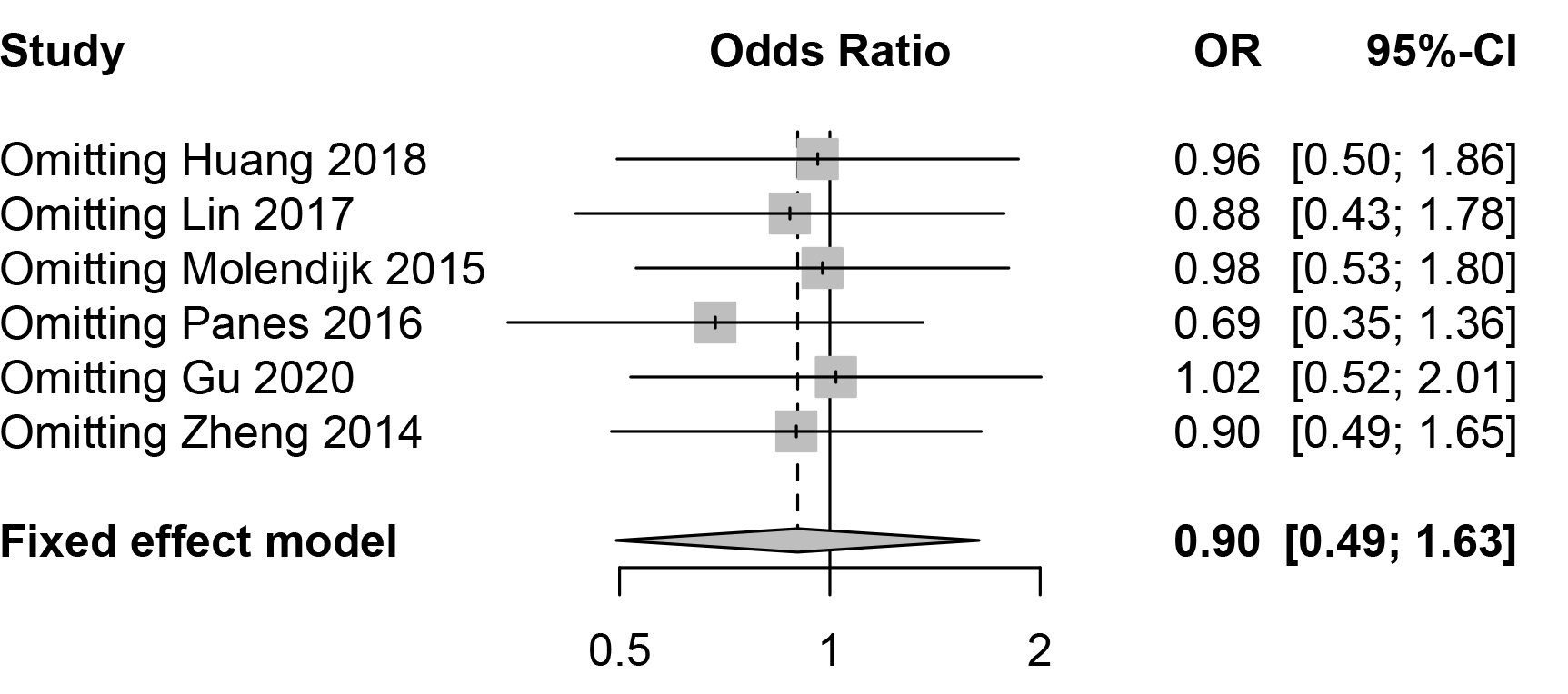

Supplement: Supplementary file 7 — Additional file 7. Leave-one-out meta-analysis for diarrhoea. [file 13287_2021_2609_MOESM7_ESM.tif]

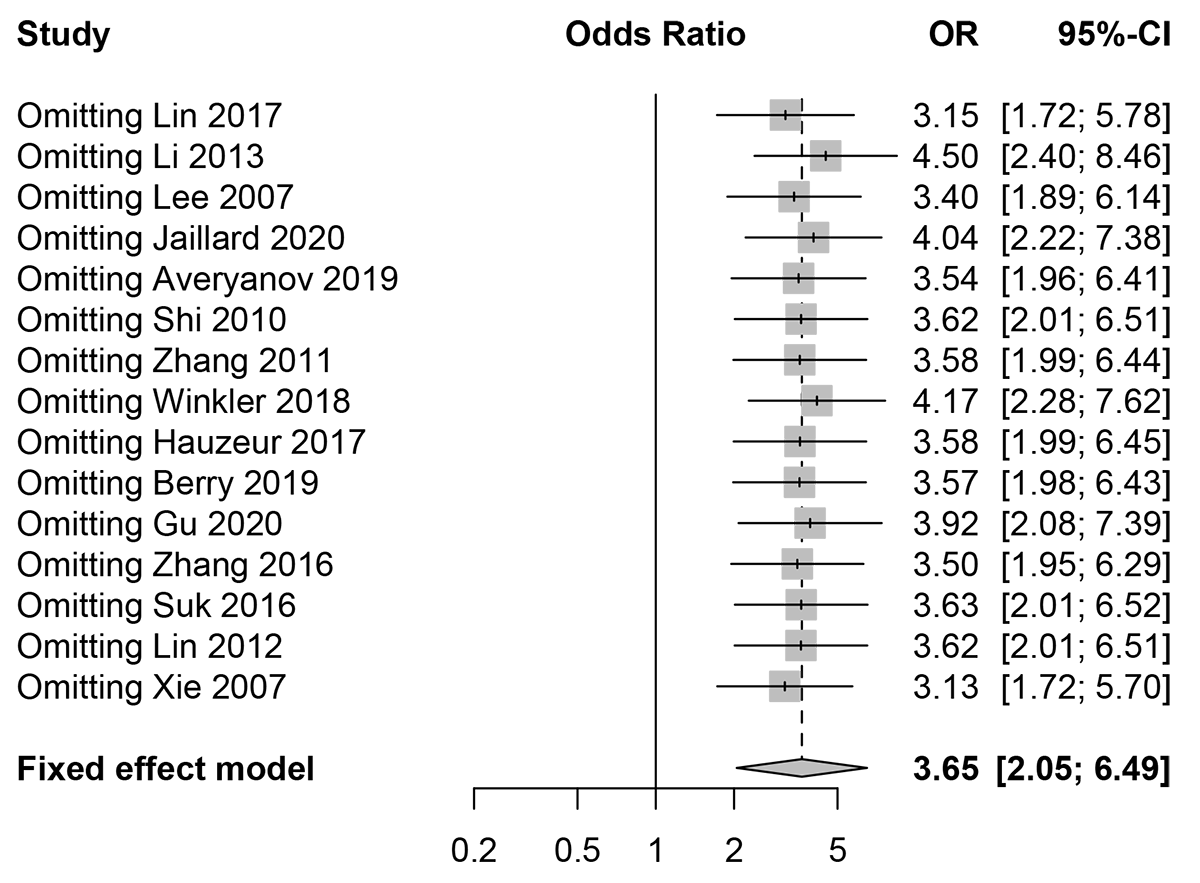

Supplement: Supplementary file 8 — Additional file 8.Leave-one-out meta-analysis for transient fever. [file 13287_2021_2609_MOESM8_ESM.tif]

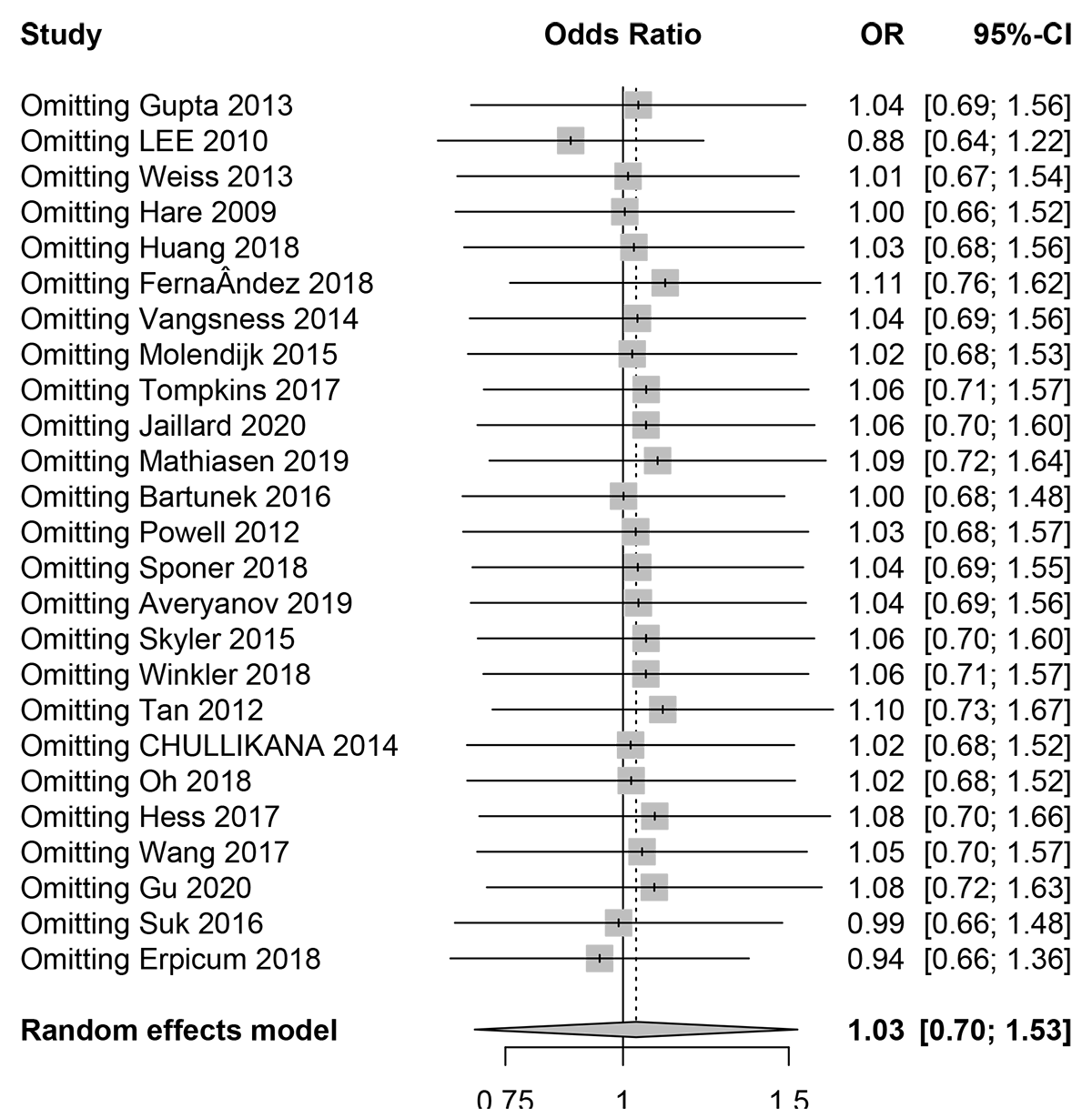

Supplement: Supplementary file 9 — Additional file 9. Leave-one-out meta-analysis for infection. [file 13287_2021_2609_MOESM9_ESM.tif]

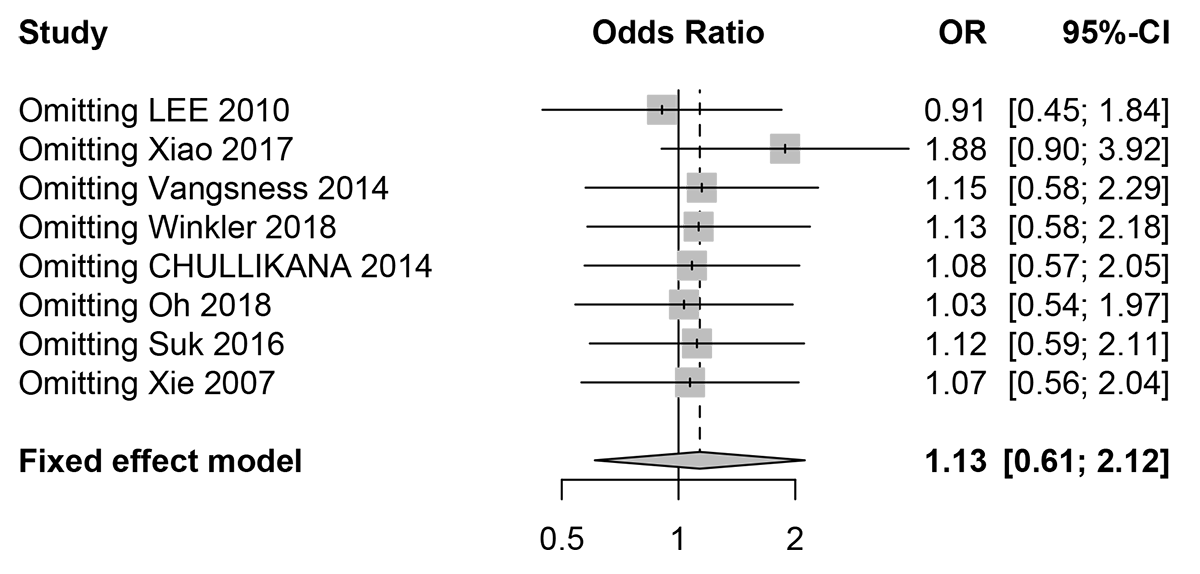

Supplement: Supplementary file 10 — Additional file 10. Leave-one-out meta-analysis for central nervous system disorders. [file 13287_2021_2609_MOESM10_ESM.tif]

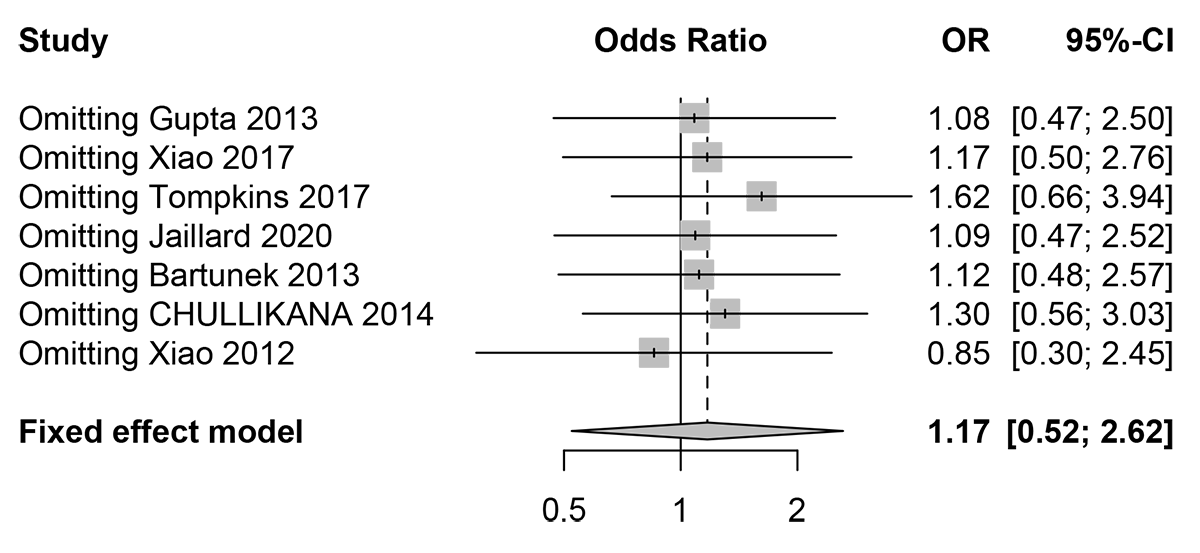

Supplement: Supplementary file 11 — Additional file 11. Leave-one-out meta-analysis for vascular disorders. [file 13287_2021_2609_MOESM11_ESM.tif]

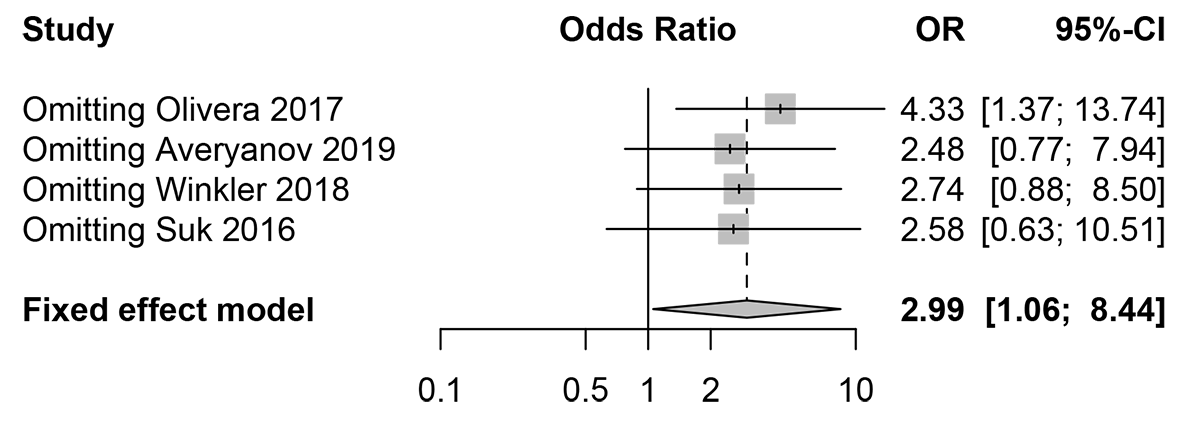

Supplement: Supplementary file 12 — Additional file 12. Leave-one-out meta-analysis for fatigue. [file 13287_2021_2609_MOESM12_ESM.tif]

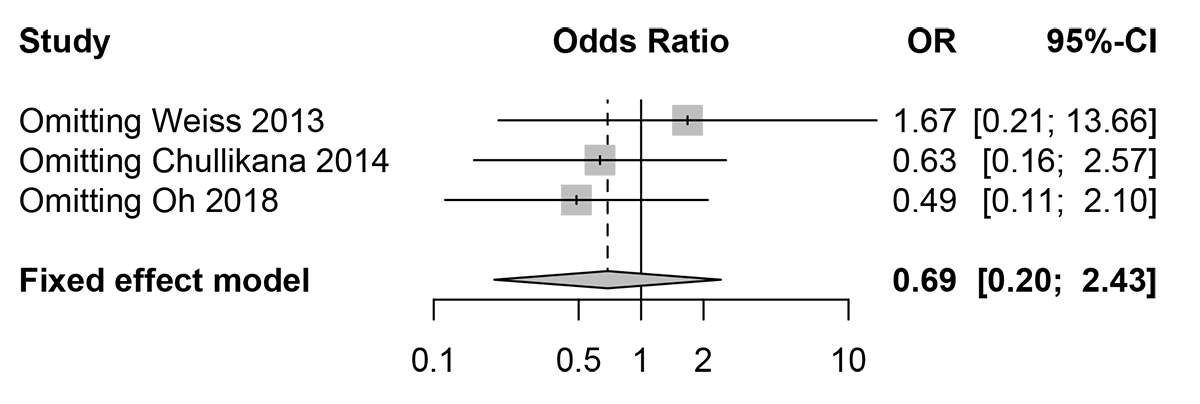

Supplement: Supplementary file 13 — Additional file 13. Leave-one-out meta-analysis for metabolism and nutrition disorders. [file 13287_2021_2609_MOESM13_ESM.tif]

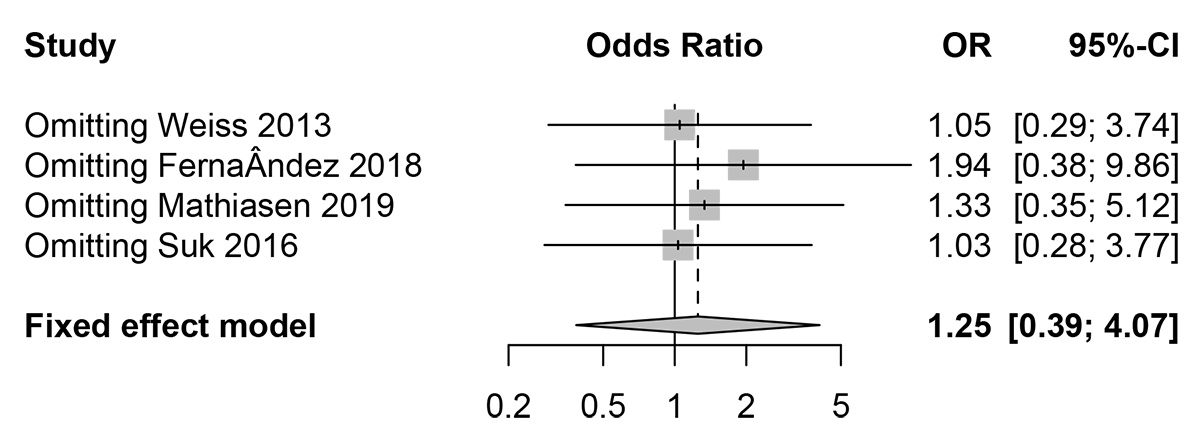

Supplement: Supplementary file 14 — Additional file 14. Leave-one-out meta-analysis for anaemia. [file 13287_2021_2609_MOESM14_ESM.tif]

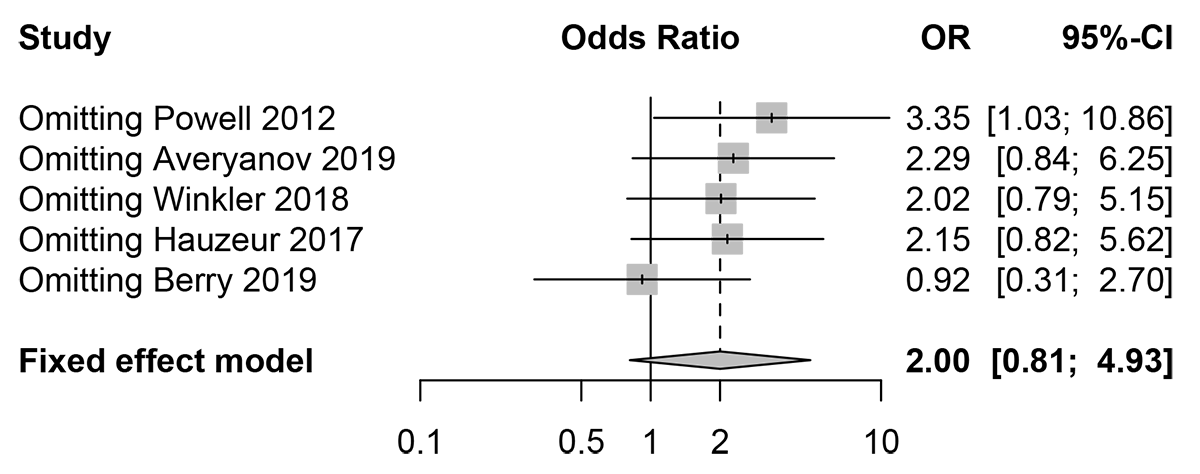

Supplement: Supplementary file 15 — Additional file 15. Leave-one-out meta-analysis for constipation. [file 13287_2021_2609_MOESM15_ESM.tif]

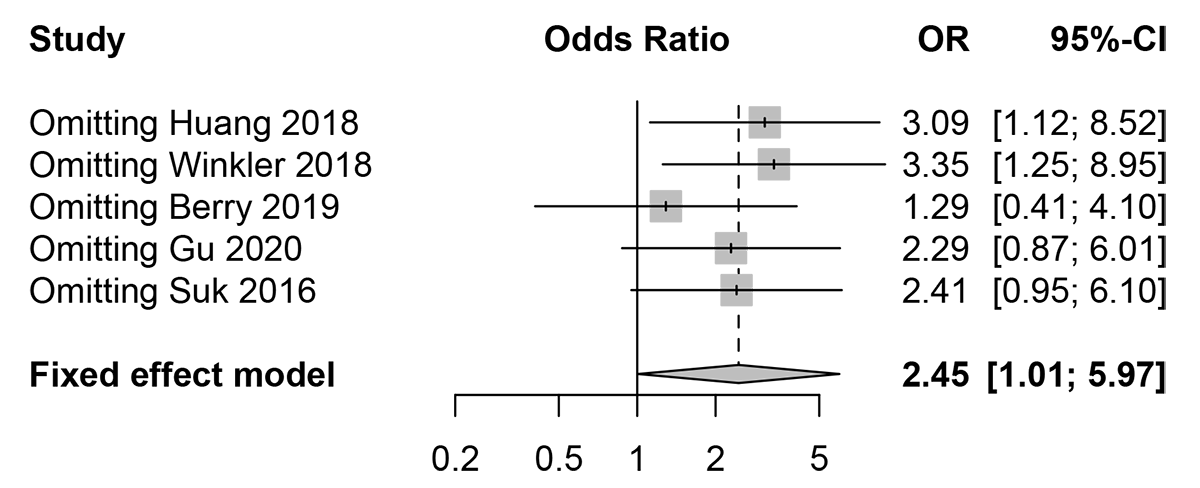

Supplement: Supplementary file 16 — Additional file 16. Leave-one-out meta-analysis for nausea. [file 13287_2021_2609_MOESM16_ESM.tif]

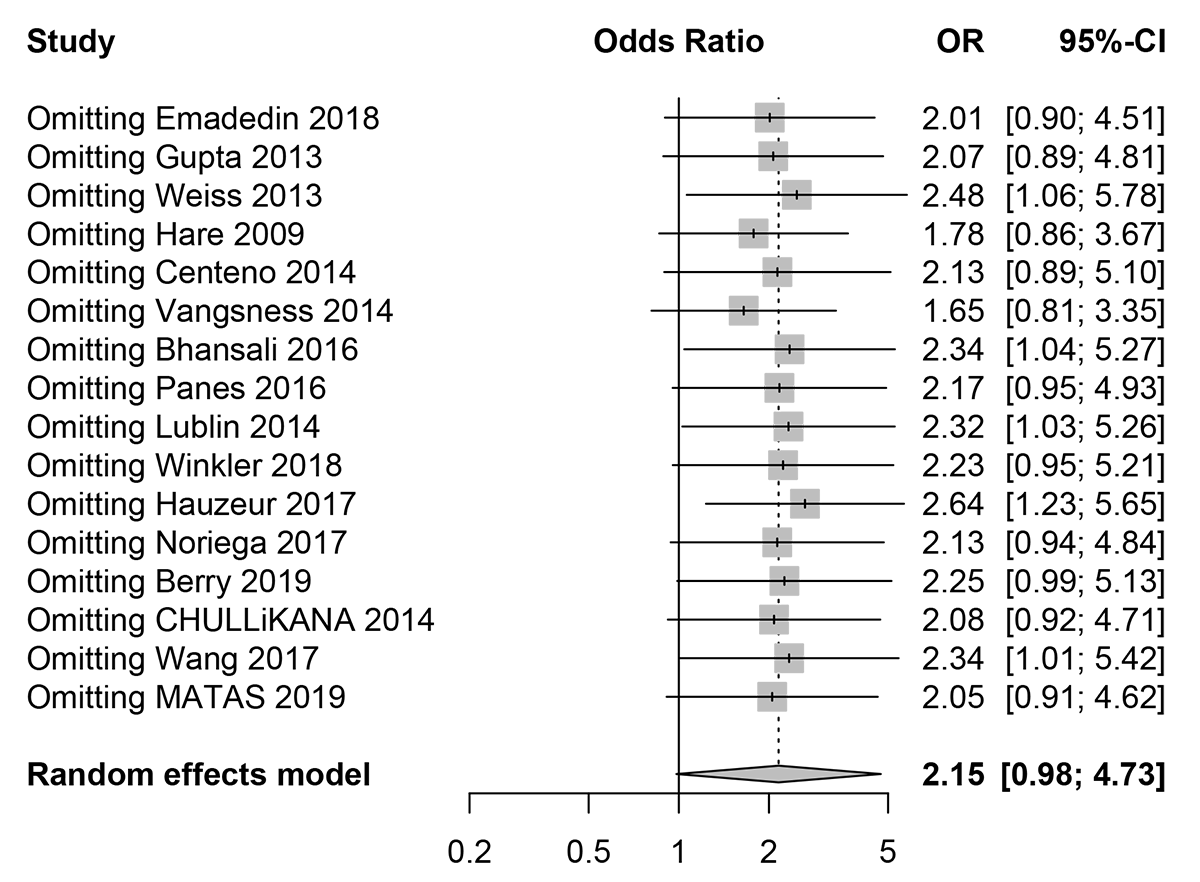

Supplement: Supplementary file 17 — Additional file 17. Leave-one-out meta-analysis of administration site adverse events in high-quality studies. [file 13287_2021_2609_MOESM17_ESM.tif]

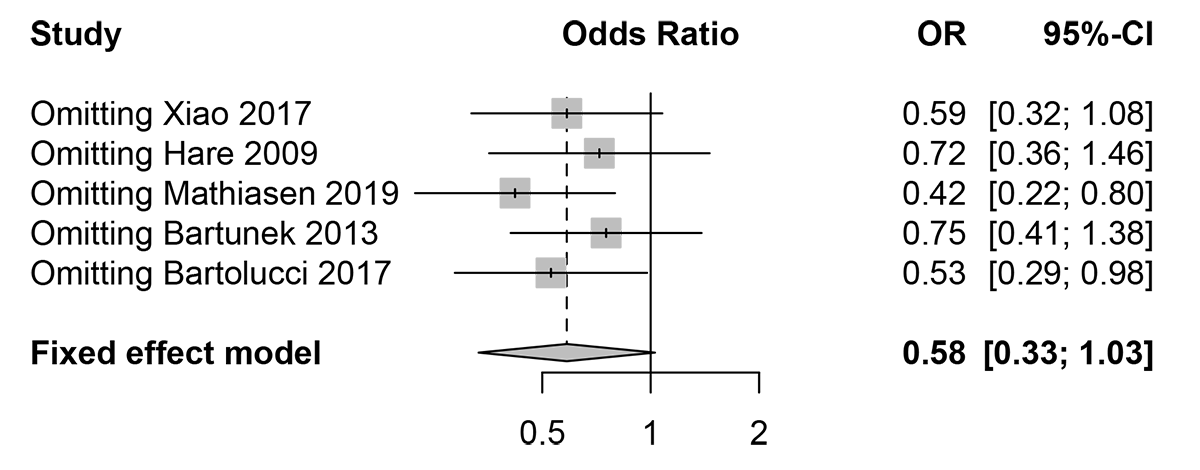

Supplement: Supplementary file 18 — Additional file 18. Leave-one-out meta-analysis for arrhythmia in high-quality studies. [file 13287_2021_2609_MOESM18_ESM.tif]

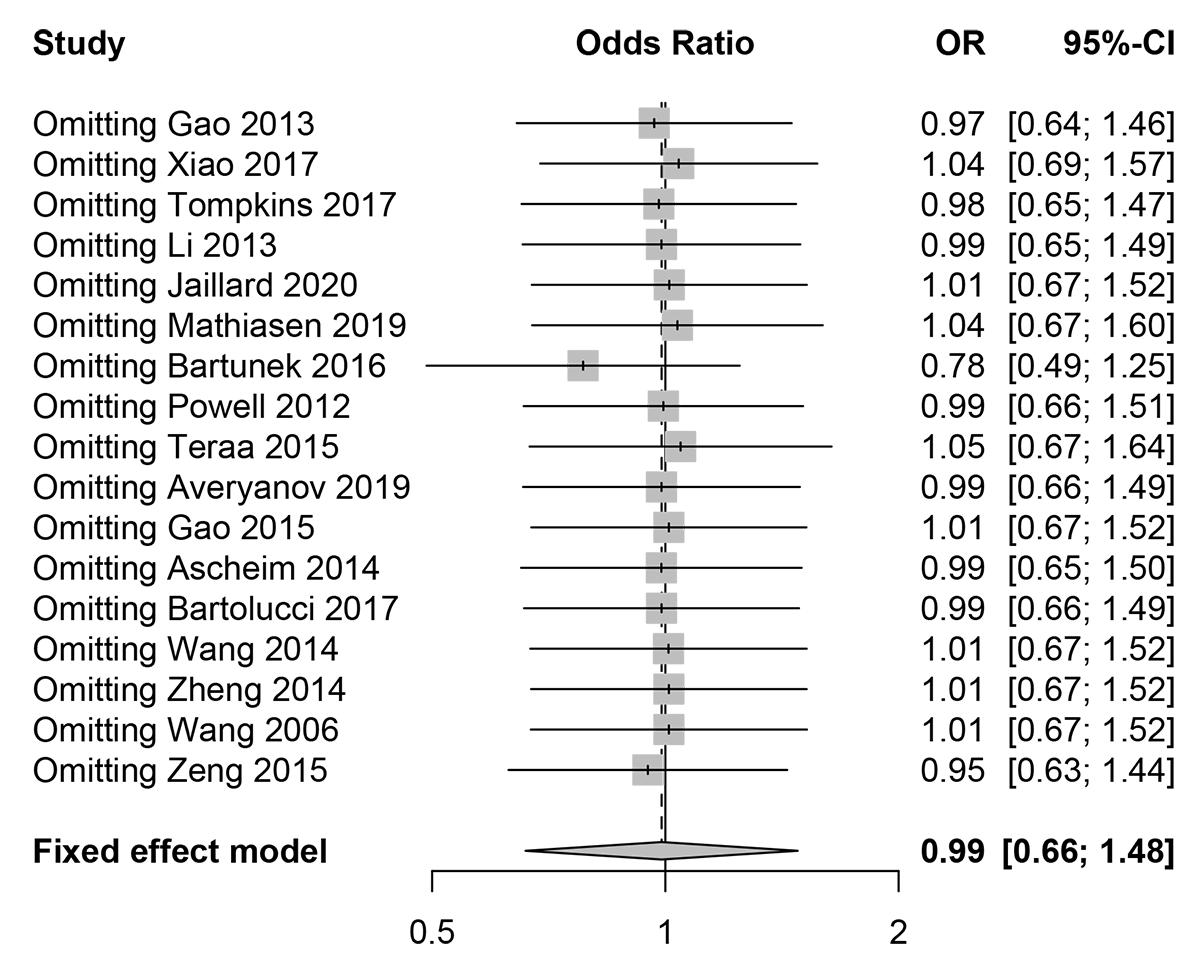

Supplement: Supplementary file 19 — Additional file 19. Leave-one-out meta-analysis for death in high-quality studies. [file 13287_2021_2609_MOESM19_ESM.tif]

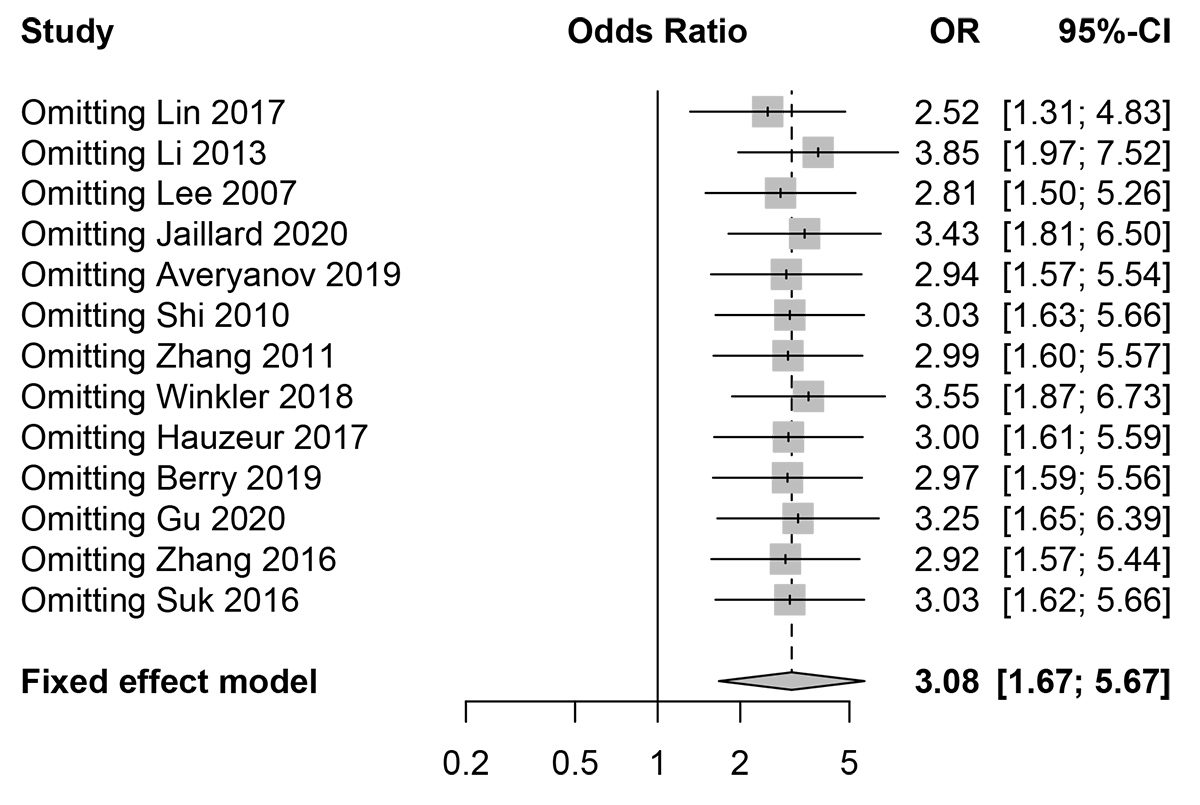

Supplement: Supplementary file 20 — Additional file 20. Leave-one-out meta-analysis for transient fever in high-quality studies. [file 13287_2021_2609_MOESM20_ESM.tif]

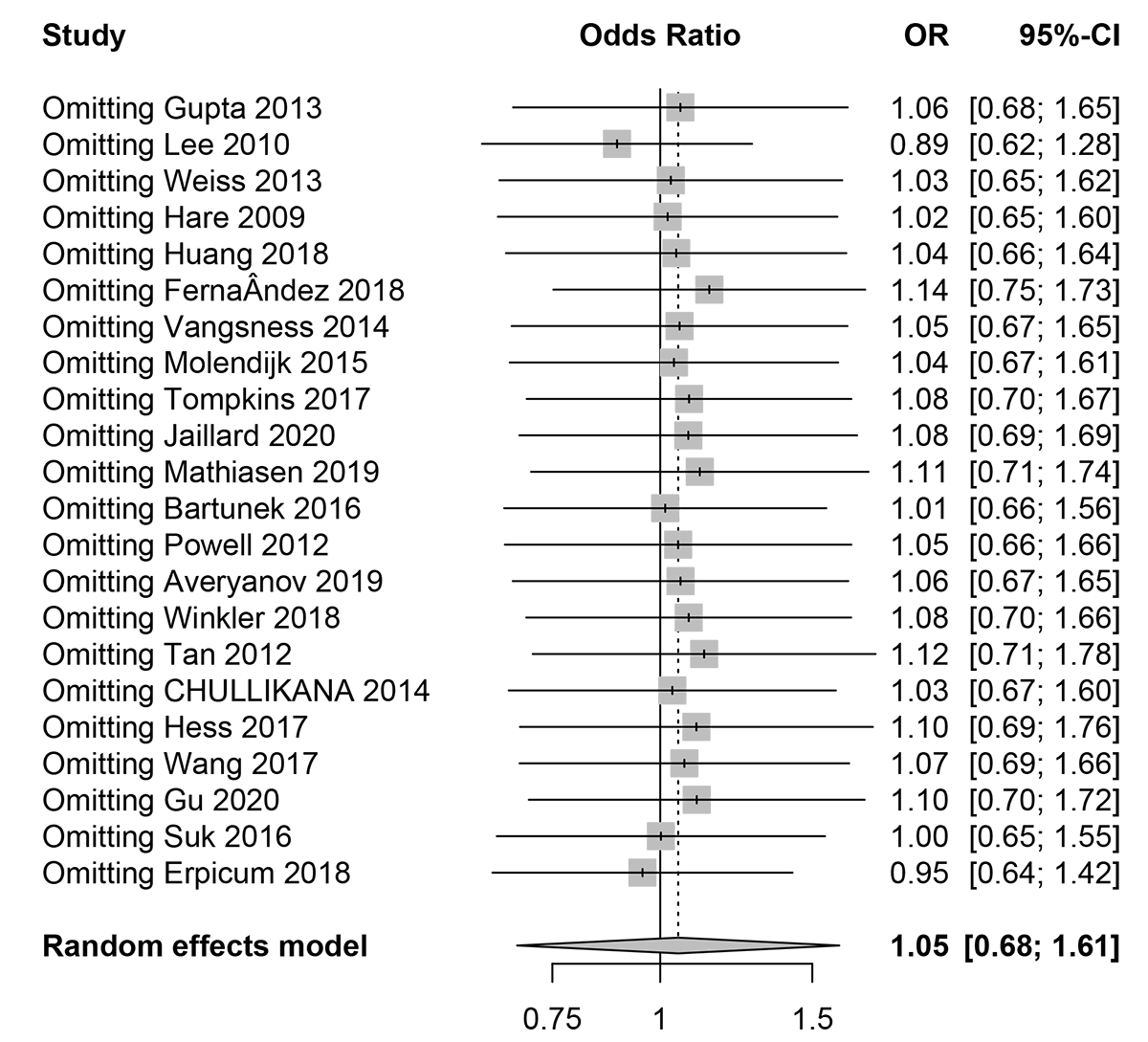

Supplement: Supplementary file 21 — Additional file 21. Leave-one-out meta-analysis for infection in high-quality studies. [file 13287_2021_2609_MOESM21_ESM.tif]

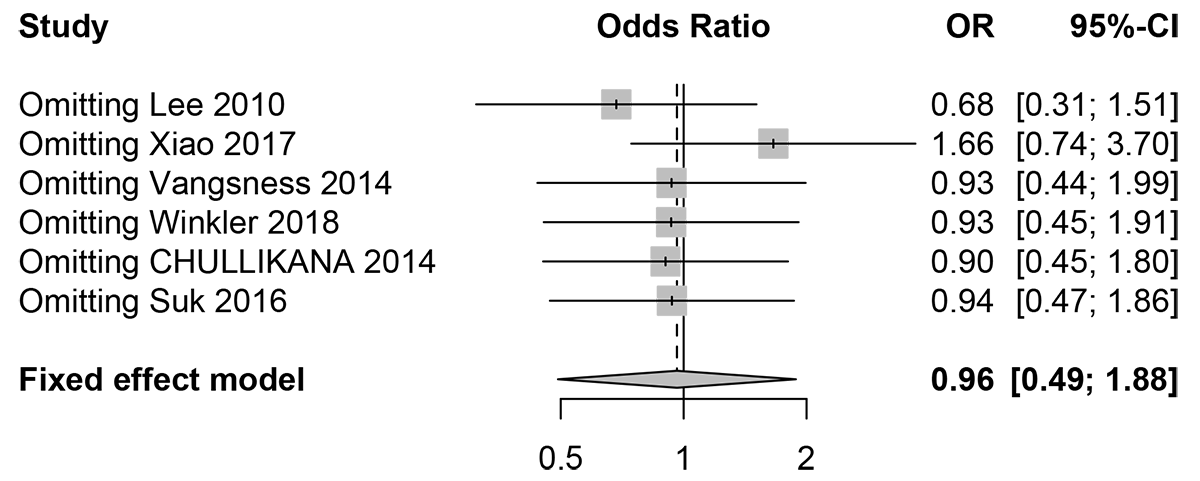

Supplement: Supplementary file 22 — Additional file 22. Leave-one-out meta-analysis of central nervous system disorders in high-quality studies. [file 13287_2021_2609_MOESM22_ESM.tif]

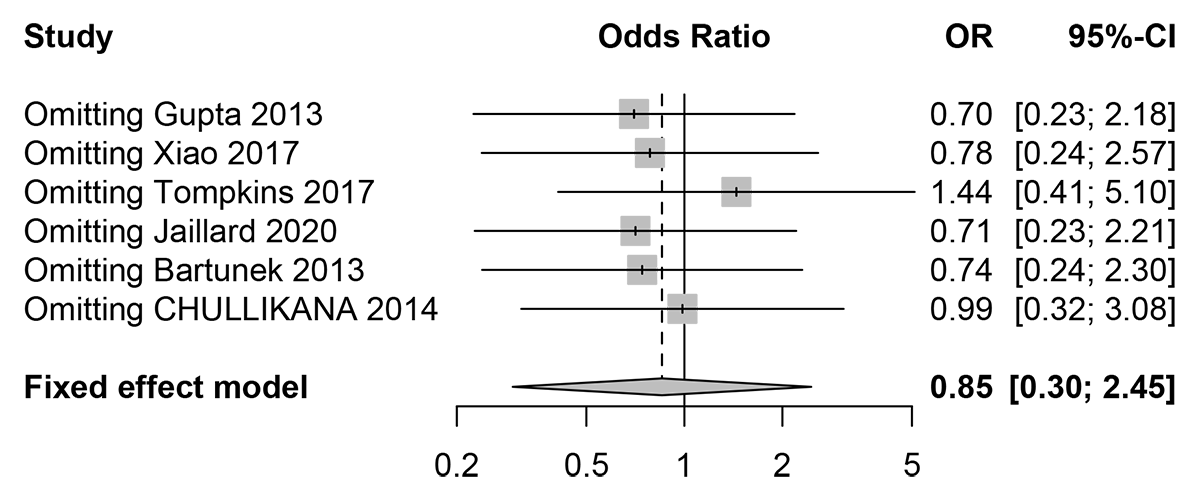

Supplement: Supplementary file 23 — Additional file 23. Leave-one-out meta-analysis for vascular disorders in high-quality studies. [file 13287_2021_2609_MOESM23_ESM.tif]

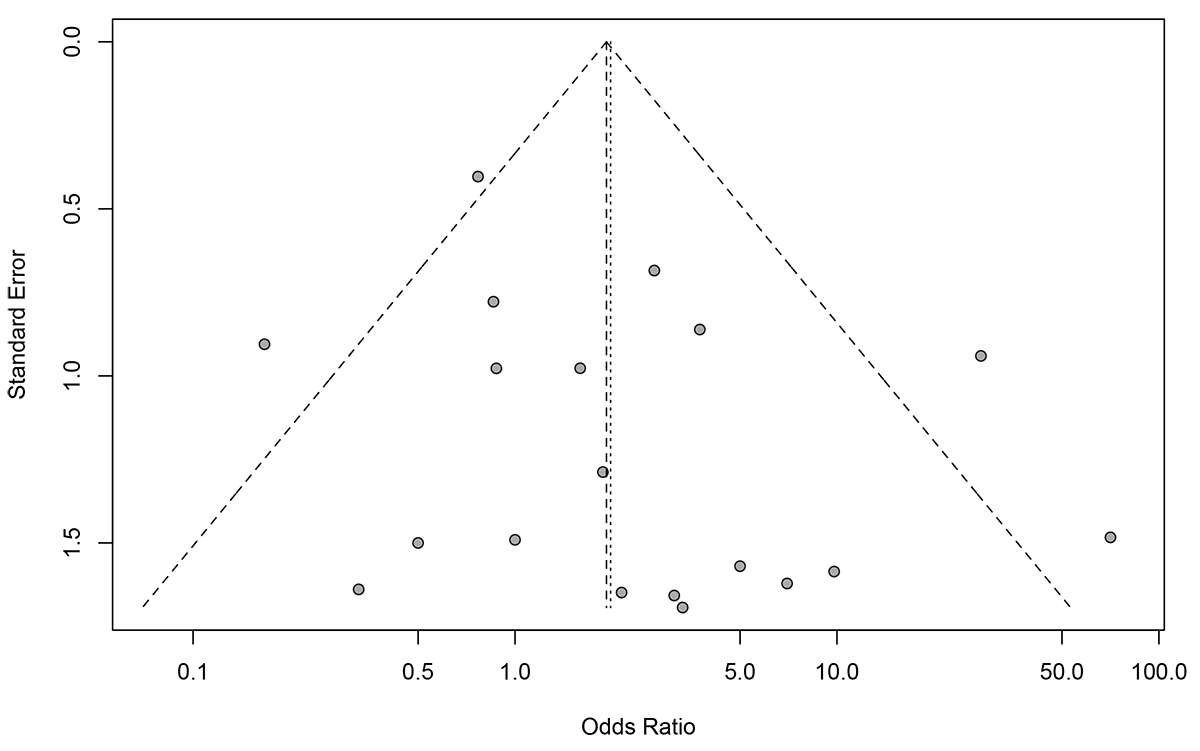

Supplement: Supplementary file 24 — Additional file 24. Funnel plot for administration site adverse events. [file 13287_2021_2609_MOESM24_ESM.tif]

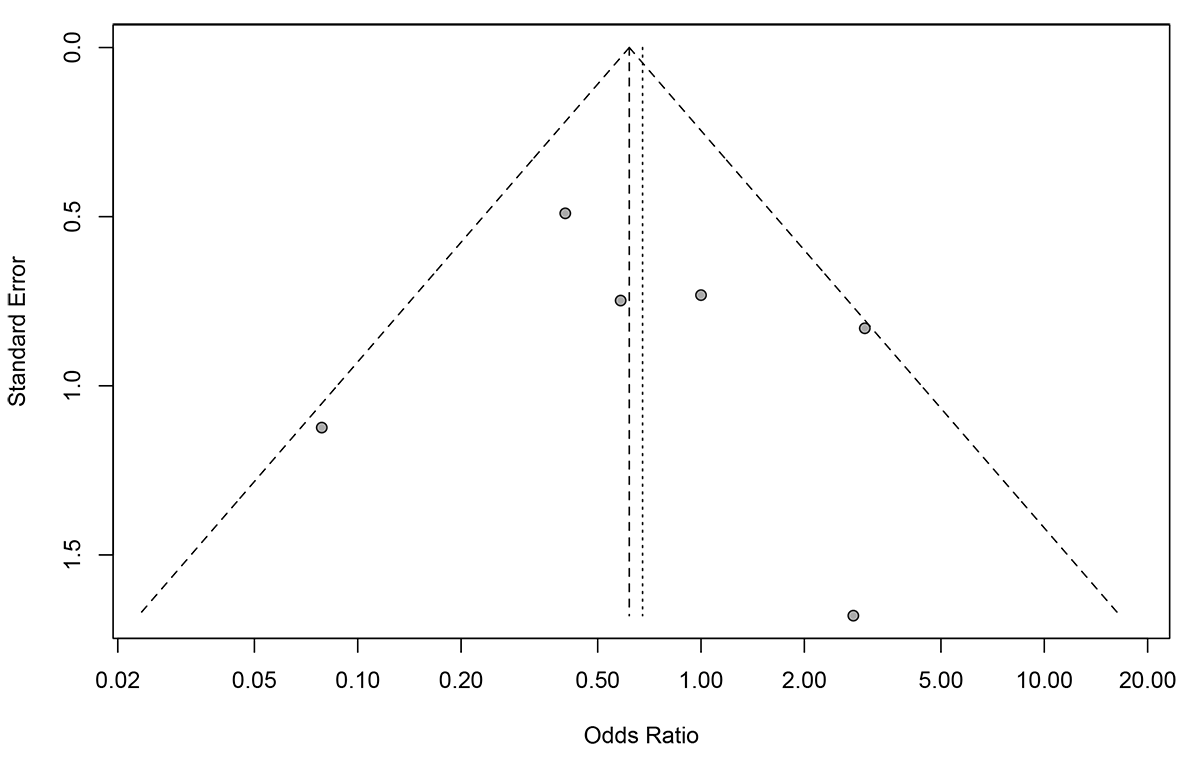

Supplement: Supplementary file 25 — Additional file 25. Funnel plot for arrhythmia. [file 13287_2021_2609_MOESM25_ESM.tif]

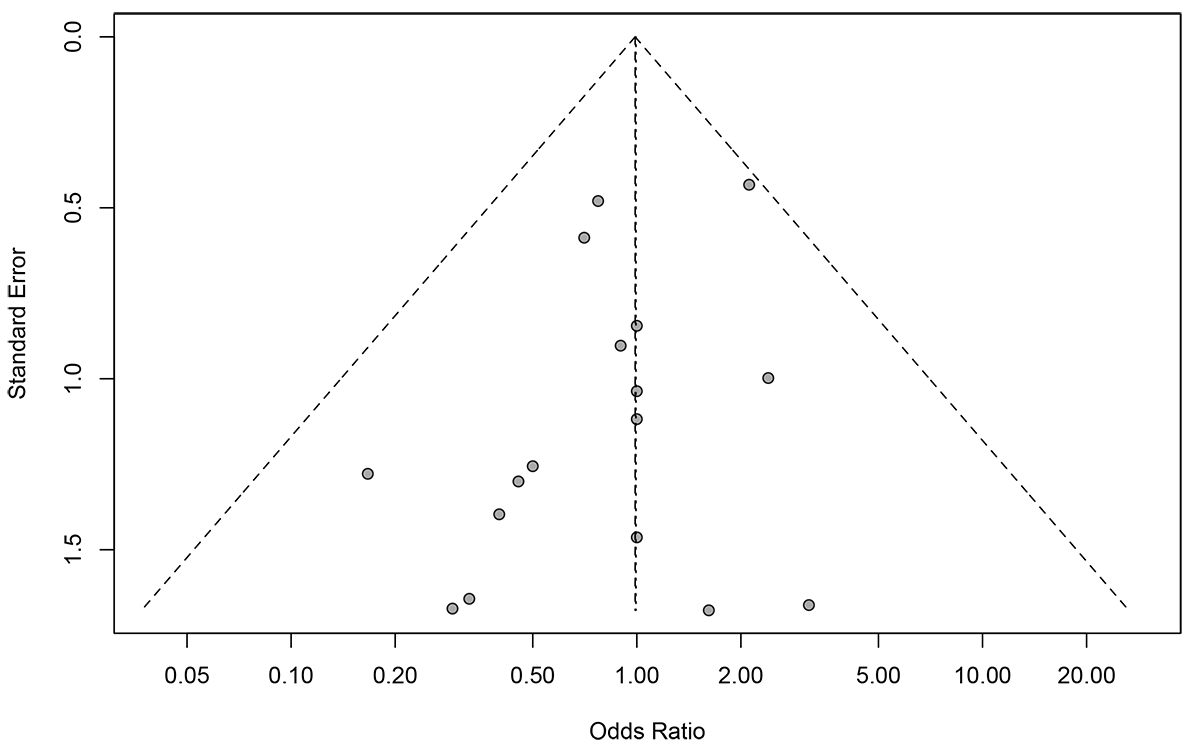

Supplement: Supplementary file 26 — Additional file 26. Funnel plot for death. [file 13287_2021_2609_MOESM26_ESM.tif]

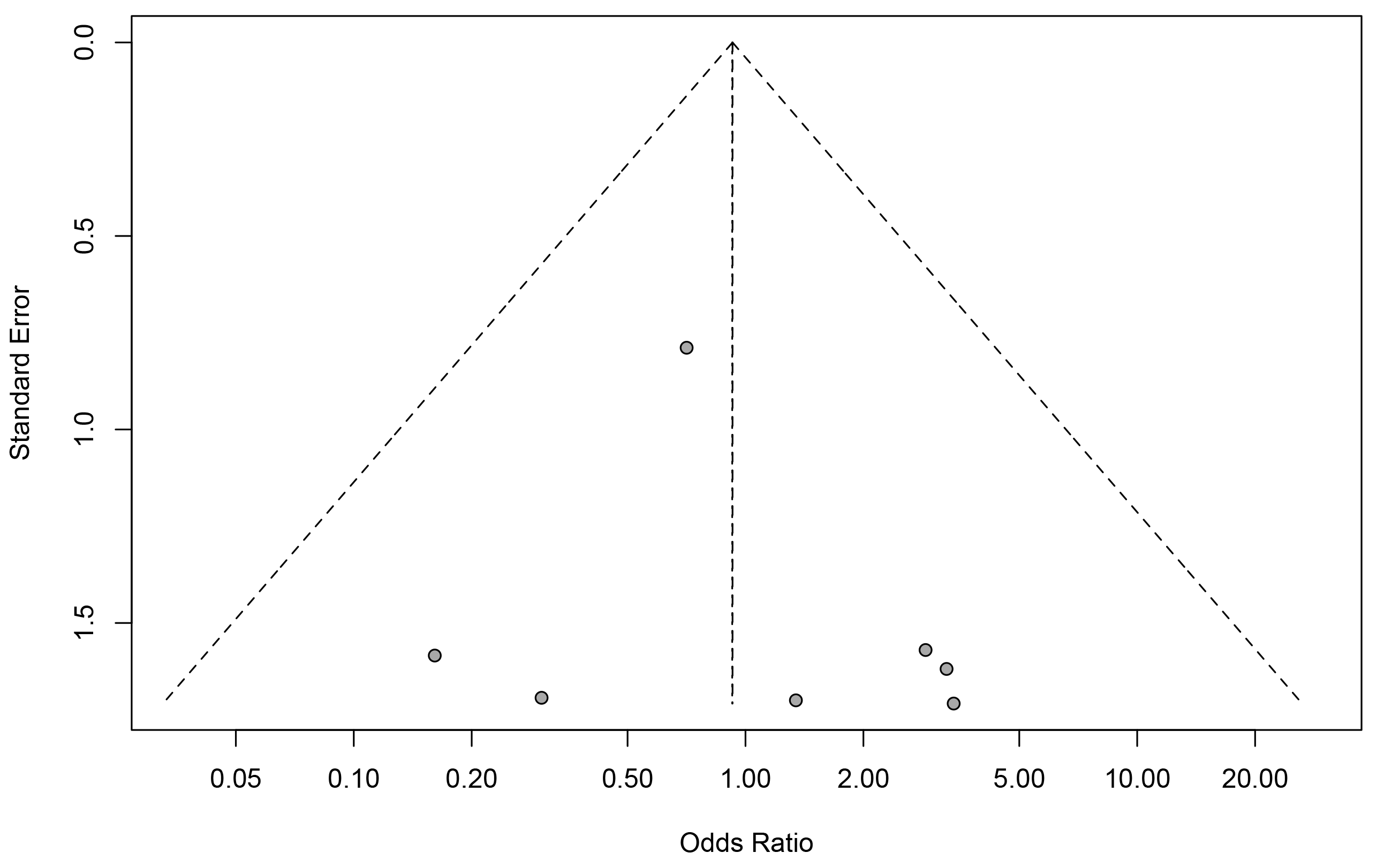

Supplement: Supplementary file 27 — Additional file 27. Funnel plot for dermatitis. [file 13287_2021_2609_MOESM27_ESM.tif]

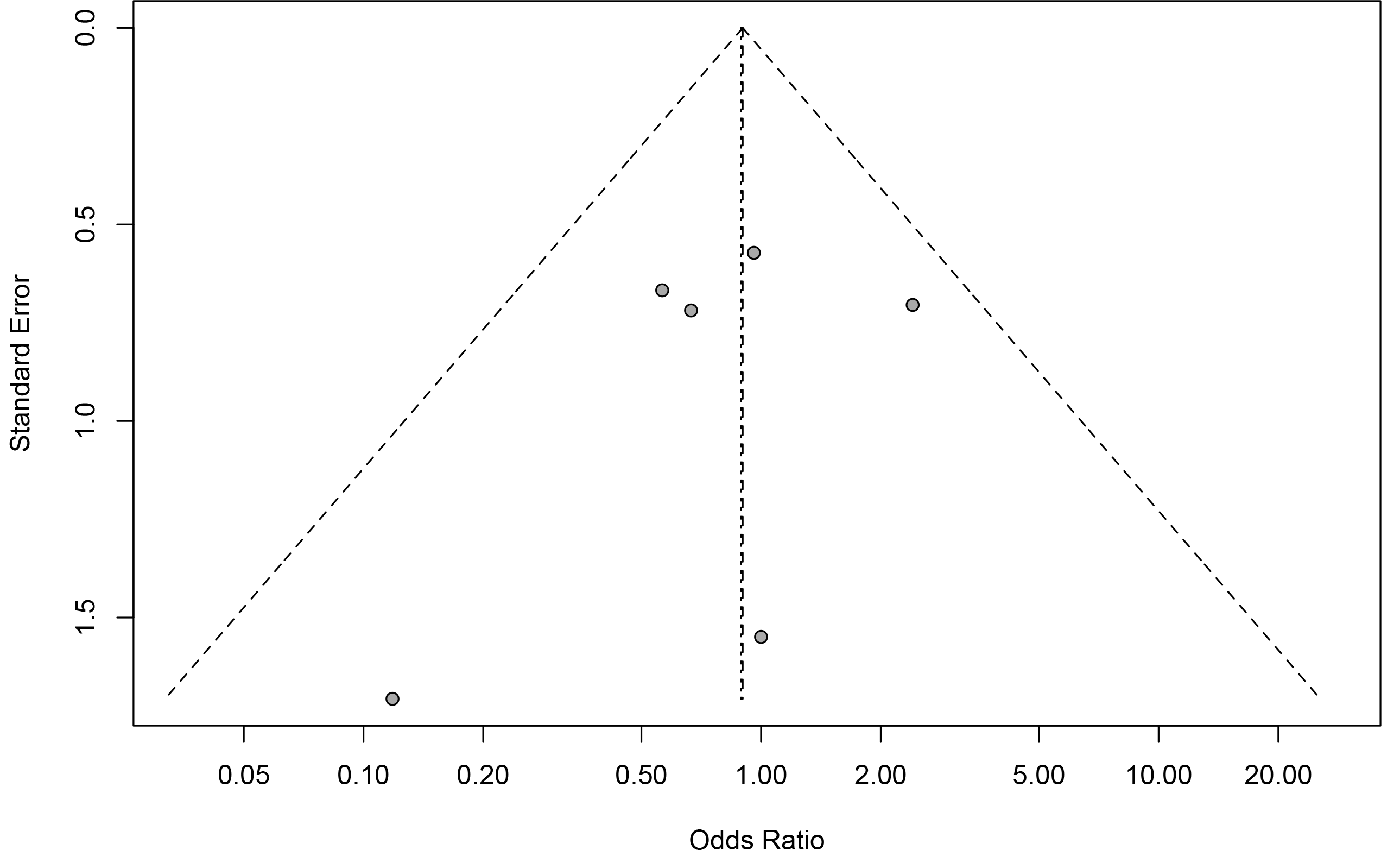

Supplement: Supplementary file 28 — Additional file 28. Funnel plot for diarrhoea. [file 13287_2021_2609_MOESM28_ESM.tif]

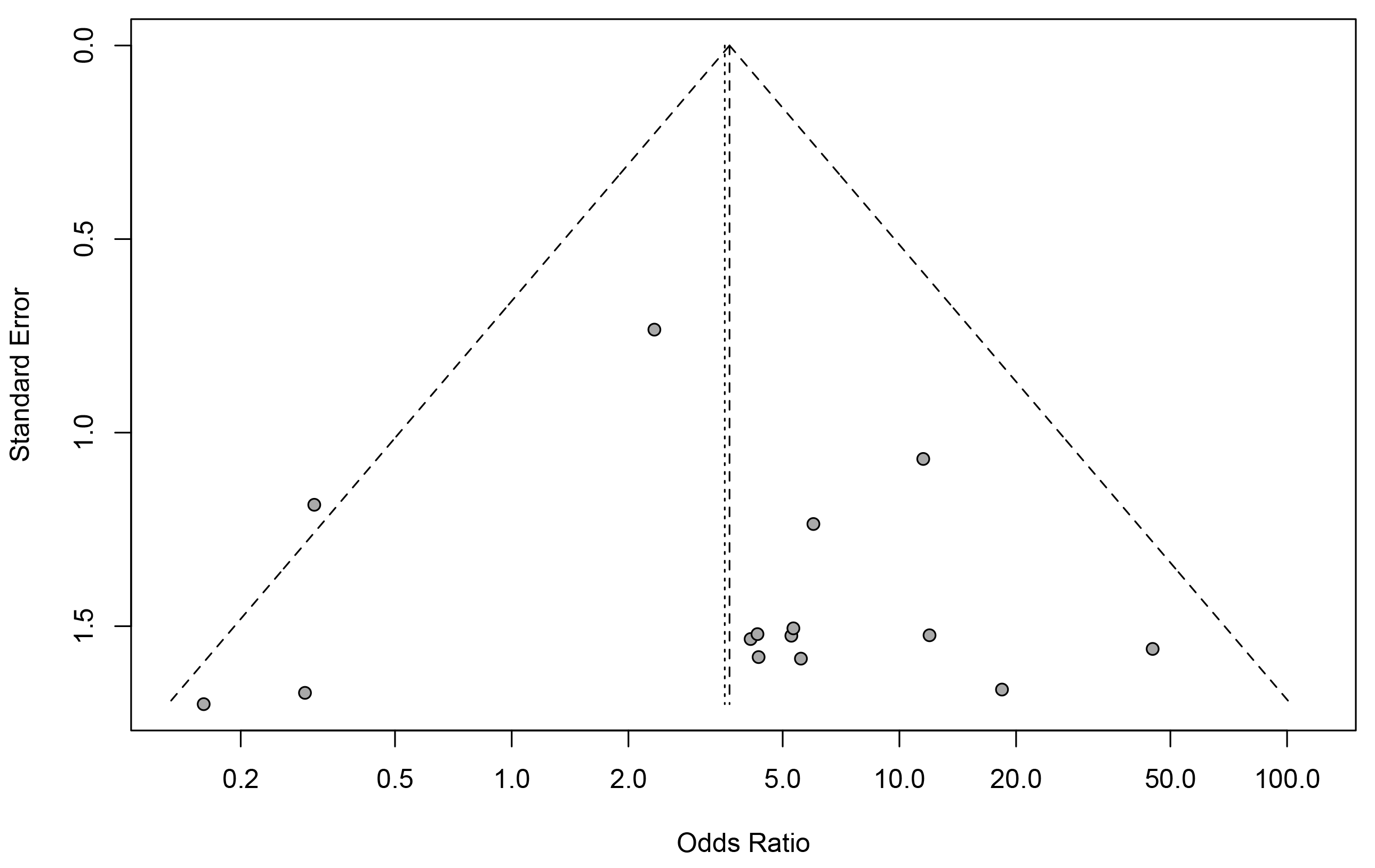

Supplement: Supplementary file 29 — Additional file 29. Funnel plot for transient fever. [file 13287_2021_2609_MOESM29_ESM.tif]

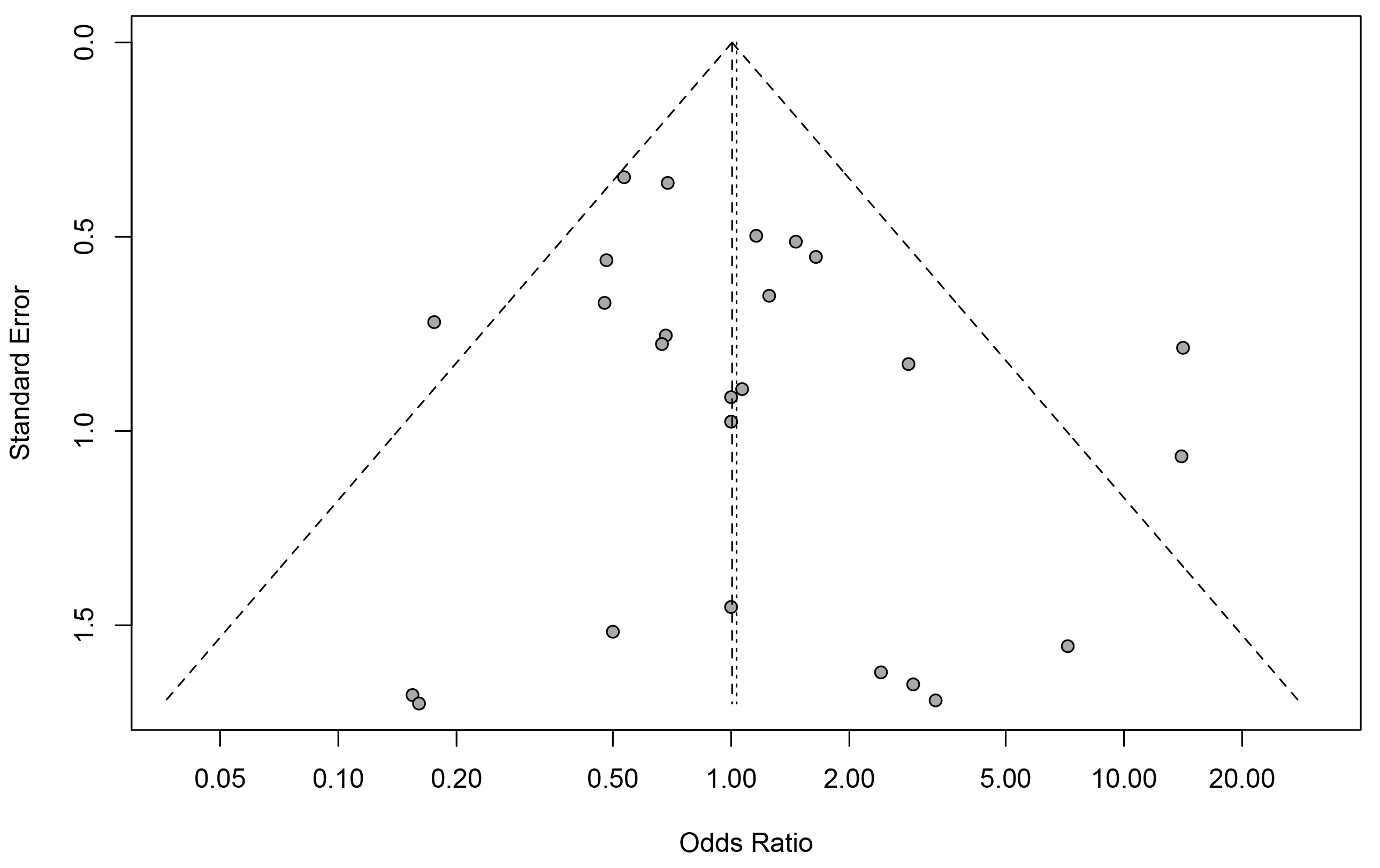

Supplement: Supplementary file 30 — Additional file 30. Funnel plot for infection. [file 13287_2021_2609_MOESM30_ESM.tif]

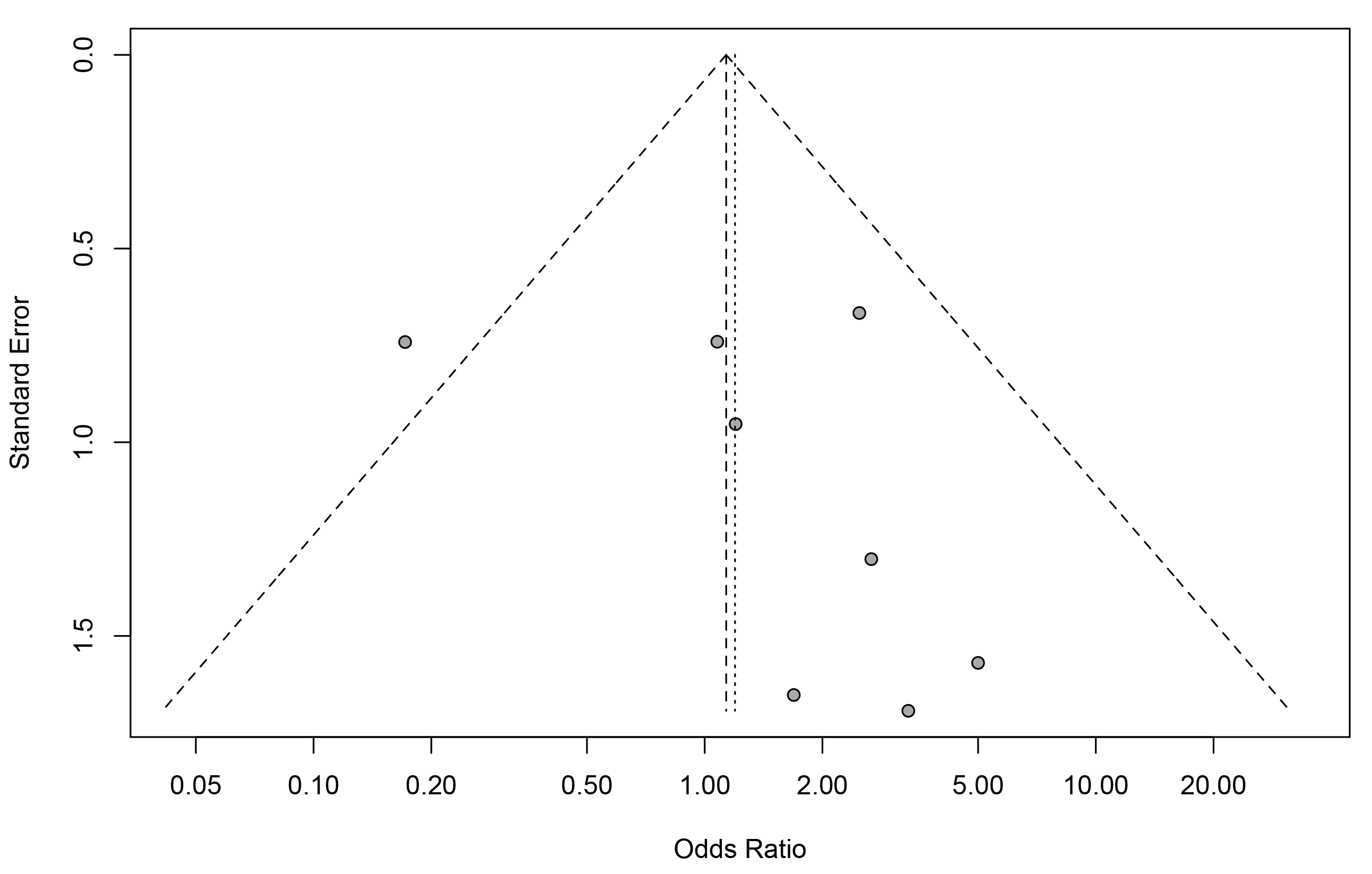

Supplement: Supplementary file 31 — Additional file 31. Funnel plot for central nervous system disorders. [file 13287_2021_2609_MOESM31_ESM.tif]

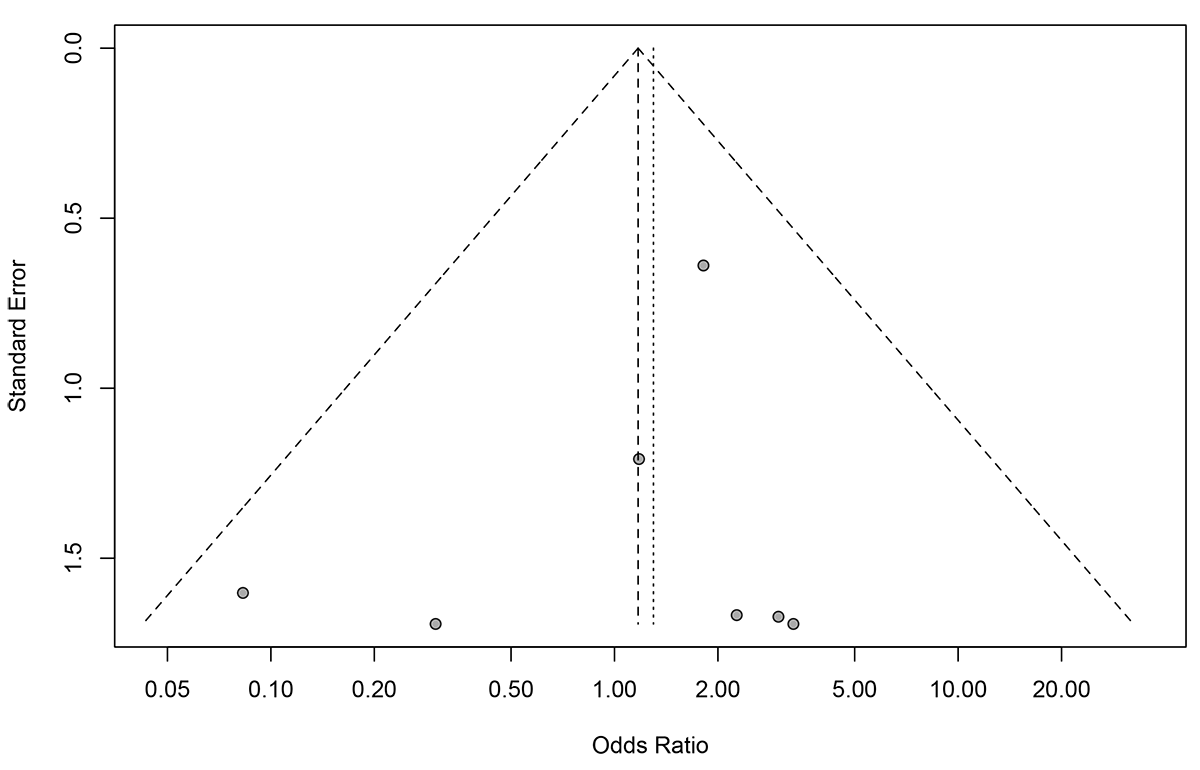

Supplement: Supplementary file 32 — Additional file 32. Funnel plot for vascular disorders. [file 13287_2021_2609_MOESM32_ESM.tif]

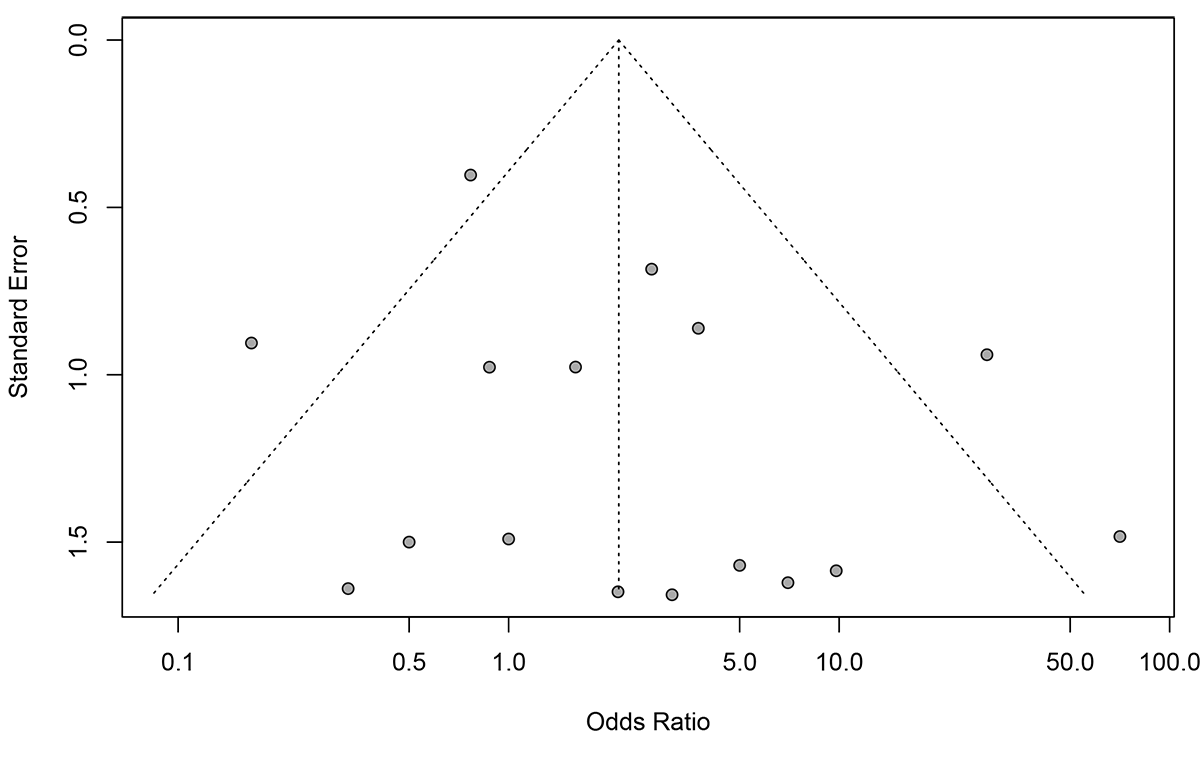

Supplement: Supplementary file 33 — Additional file 33. Funnel plot for administration site adverse events in high-quality studies. [file 13287_2021_2609_MOESM33_ESM.tif]

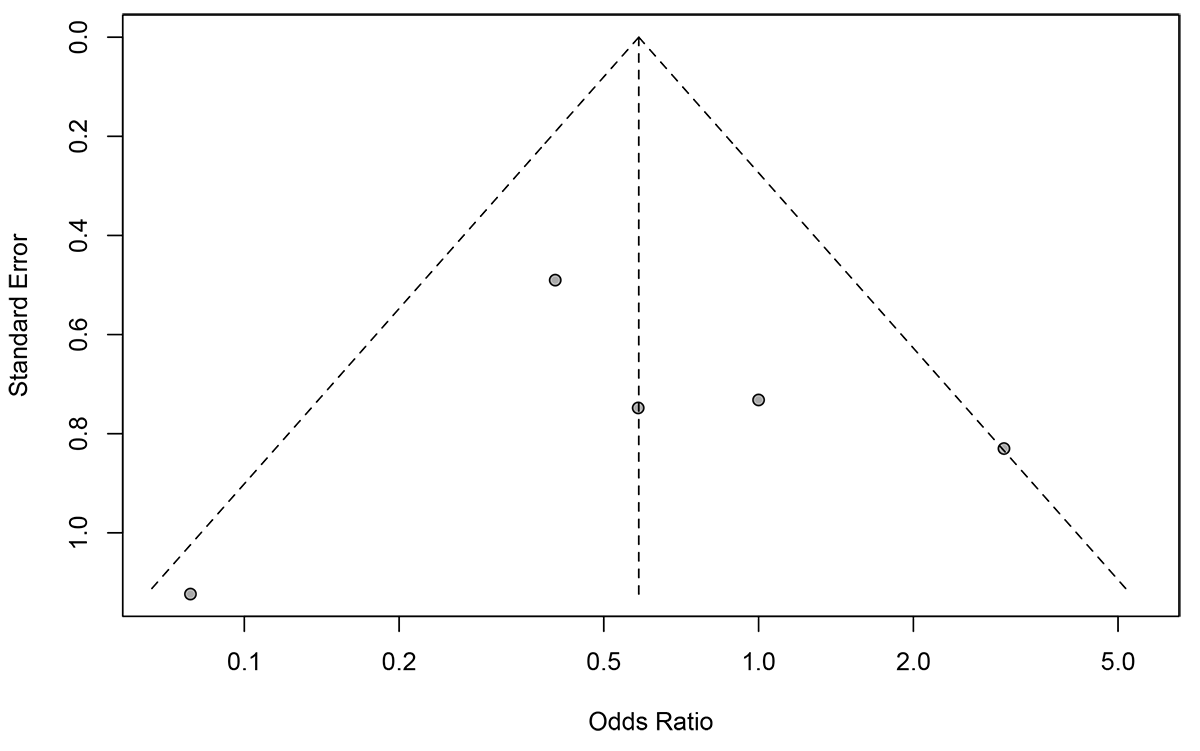

Supplement: Supplementary file 34 — Additional file 34. Funnel plot for arrythmia in high-quality studies. [file 13287_2021_2609_MOESM34_ESM.tif]

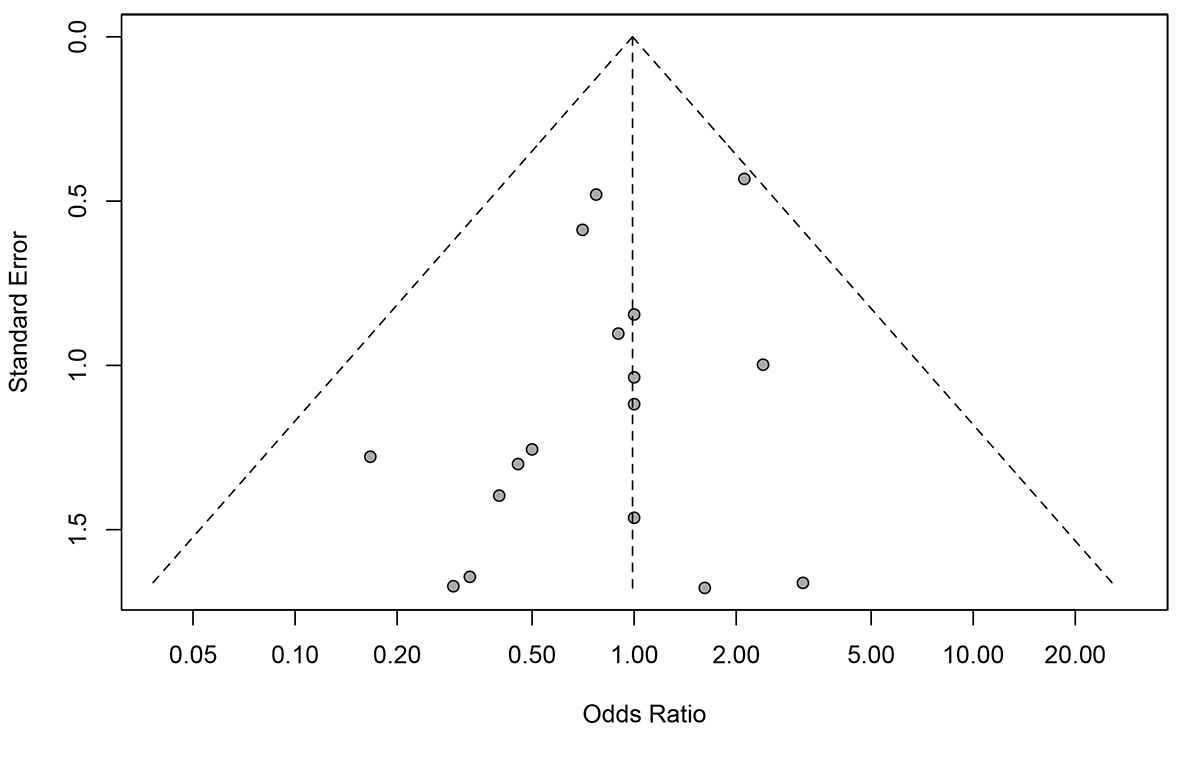

Supplement: Supplementary file 35 — Additional file 35. Funnel plot for death in high-quality studies. [file 13287_2021_2609_MOESM35_ESM.tif]

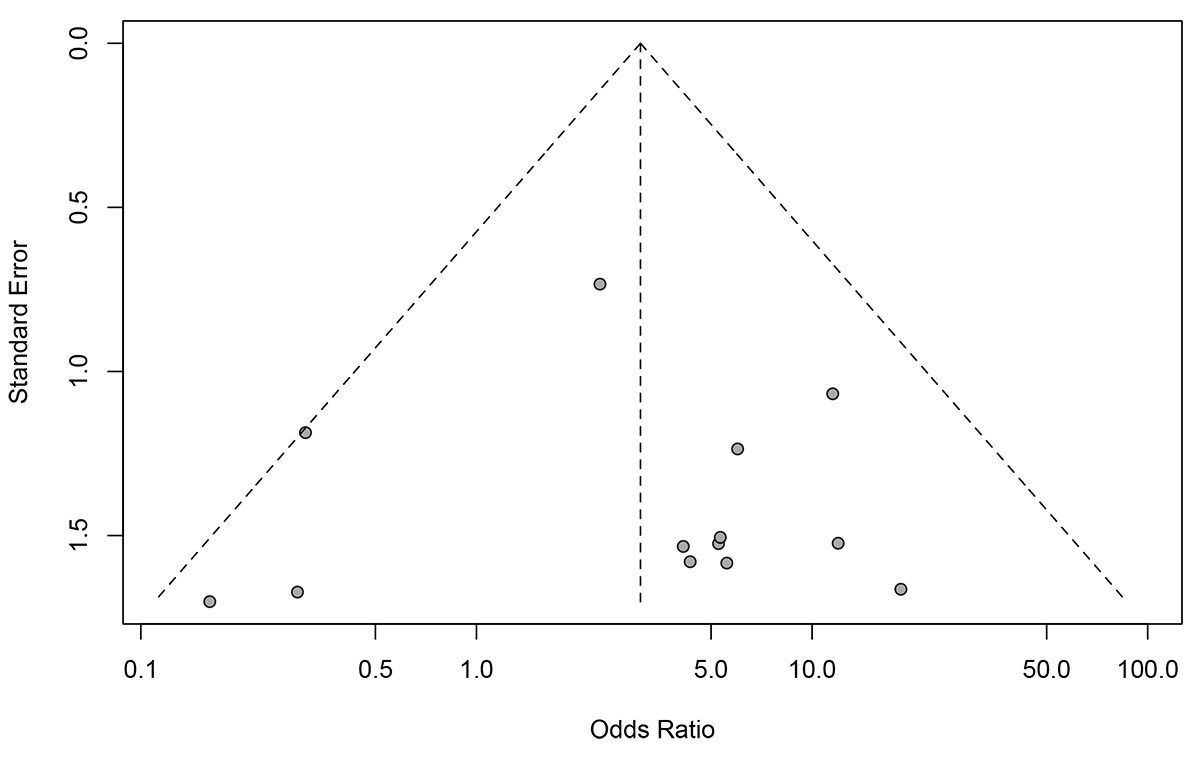

Supplement: Supplementary file 36 — Additional file 36. Funnel plot for fever in high-quality studies. [file 13287_2021_2609_MOESM36_ESM.tif]

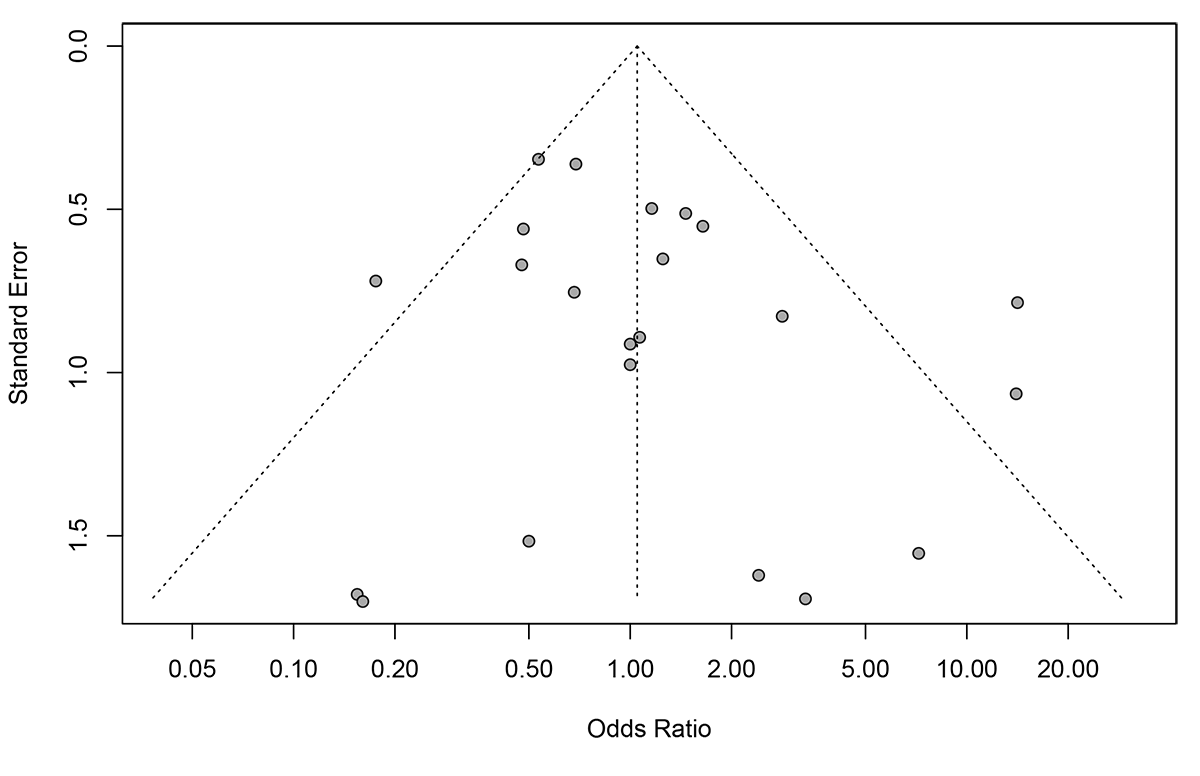

Supplement: Supplementary file 37 — Additional file 37. Funnel plot for infection in high-quality studies. [file 13287_2021_2609_MOESM37_ESM.tif]

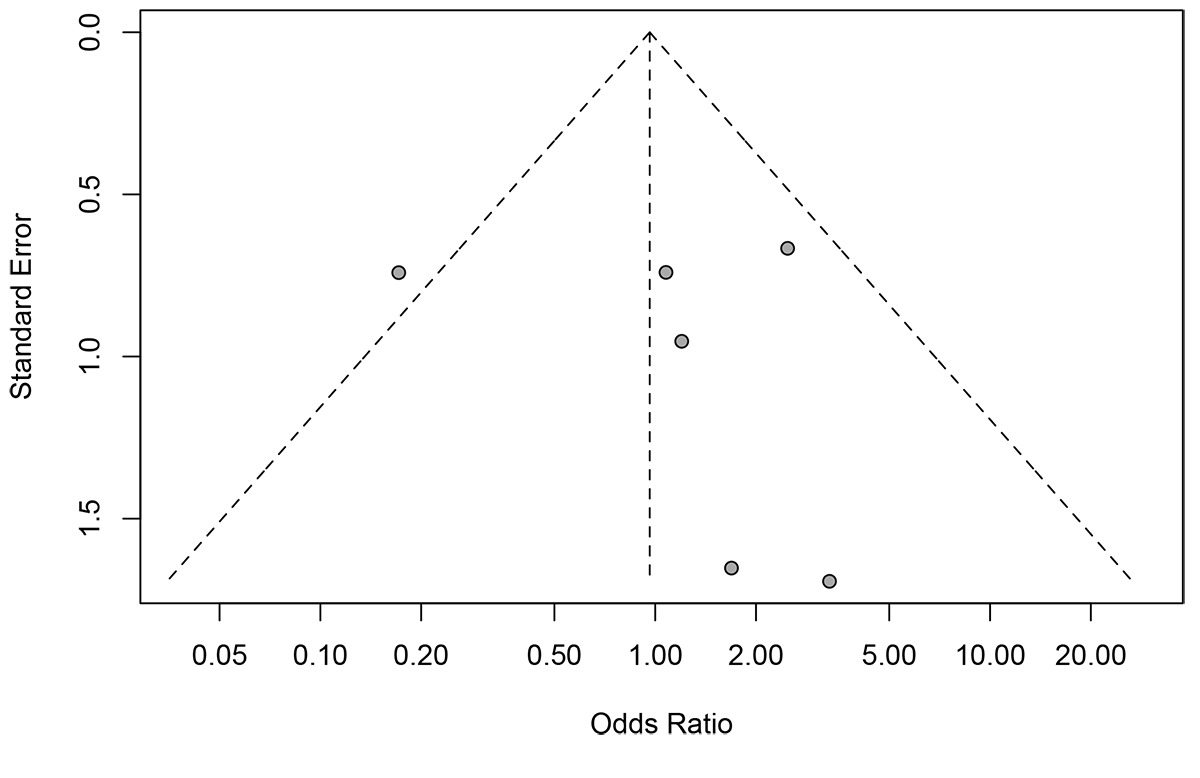

Supplement: Supplementary file 38 — Additional file 38. Funnel plot for central nervous system disorders in high-quality studies. [file 13287_2021_2609_MOESM38_ESM.tif]

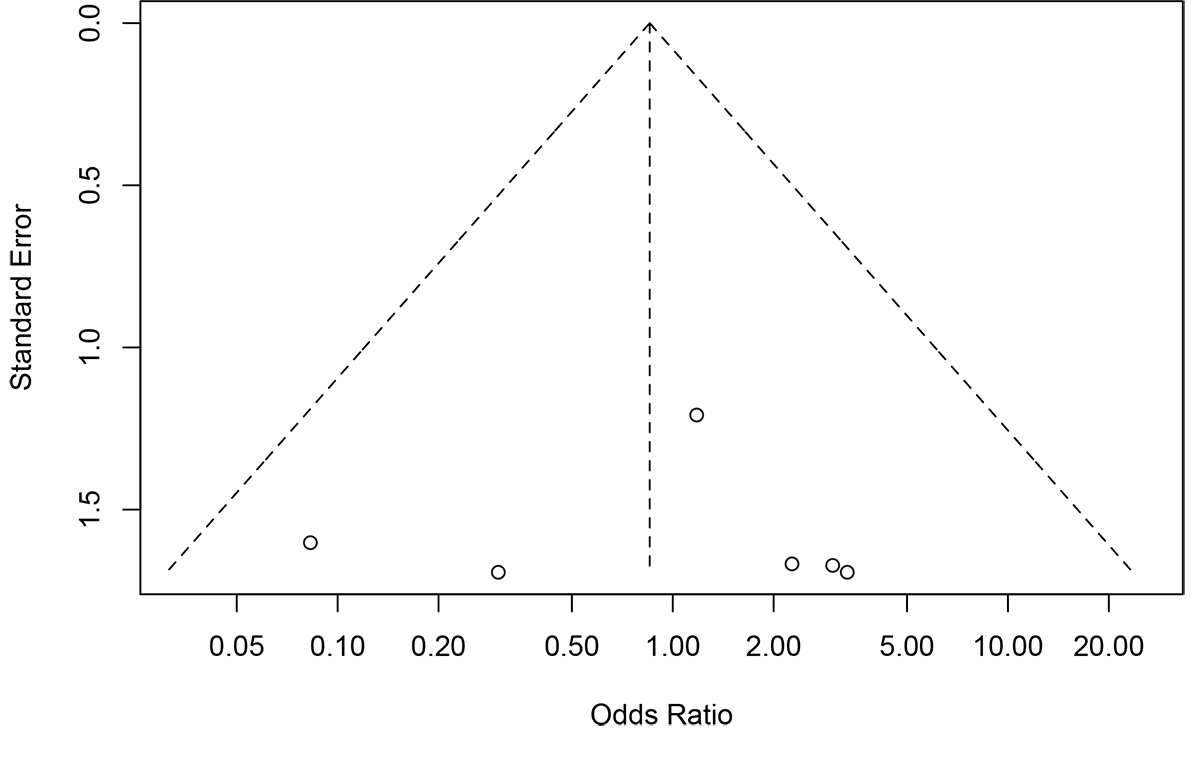

Supplement: Supplementary file 39 — Additional file 39. Funnel plot for vascular disorders in high-quality studies. [file 13287_2021_2609_MOESM39_ESM.tif]
